# Cost-effectiveness analysis of sacituzumab tirumotecan vs. chemotherapy for patients with EGFR-TKI–resistant, EGFR-mutated advanced non-small cell lung cancer in China

**DOI:** 10.3389/fpubh.2026.1843636

**Published:** 2026-07-27

**Authors:** Kai Xu, Yuting Yan, Zhaoliu Cao, Jinchun Liu, Hongting Yao, Yuyang Sun, Jingyu Zhang, Hong Wu

**Affiliations:** 1 Department of Pharmacy, Nanjing Qixia Community Healthcare Center, Nanjing, Jiangsu, China; 2 Shanghai Key Laboratory of Emotions and Affective Disorders, Songjiang District Central Hospital, Songjiang Research Institute, Shanghai Jiao Tong University School of Medicine, Shanghai, China; 3 Department of Pharmacy, Nanjing Qixia District Hospital, Nanjing, Jiangsu, China; 4 Department of Pharmacy, Nanjing Drum Tower Hospital, Nanjing, Jiangsu, China; 5 Department of Pharmaceutical Regulatory Science and Pharmacoeconomics, School of Pharmacy, Nanjing Medical University, Nanjing, Jiangsu, China; 6 Department of Oncology, Affiliated Lianyungang Clinical College of Nantong University, Lianyungang, Jiangsu, China; 7 Department of Pharmacy, The Second People's Hospital of Lianyungang & The Oncology Hospital of Lianyungang, Lianyungang, Jiangsu, China; 8 The Second People's Hospital of Lianyungang, Affiliated to Kangda College of Nanjing Medical University, Lianyungang, Jiangsu, China

**Keywords:** cost-effectiveness, non-small cell lung cancer, OptiTROP-Lung04 trial, partitioned survival model, sacituzumab tirumotecan

## Abstract

**Background:**

In the OptiTROP-Lung04 trial, sacituzumab tirumotecan (sac-TMT) yielded a median progression-free survival of 8.3 months—a clinically significant outcome that has approval by the National Medical Products Administration. This study evaluates the cost-effectiveness of sac-TMT as a later-line therapy for patients with EGFR-mutant advanced NSCLC who have progressed on EGFR-TKIs, from the perspective of Chinese healthcare system.

**Methods:**

A partitioned survival model was constructed to simulate patients' health state transitions in 28-day cycles and predict long-term survival and economic outcomes over a 10-year time horizon for the two treatment strategies. Clinical data were sourced from the OptiTROP-Lung04 trial, whereas cost and utility parameters were extracted from local medical charges and published literatures. The model calculated total costs, life-years (LYs), quality-adjusted life-years (QALYs), and the incremental cost-effectiveness ratio (ICER). One-way and probabilistic sensitivity analyses were conducted to verify the robustness of model outputs. Scenario analyses evaluated changes in the ICER under different sac-TMT price levels and alternative time horizons. Subgroup analyses estimated ICER values corresponding to hazard ratios across distinct subgroups.

**Results:**

Compared with the chemotherapy group, sac-TMT incurred an incremental cost of $28,285.28 and generated an additional 0.84 QALYs and 1.74 LYs, yielding an ICER of $33,514.79/QALY. One-way sensitivity analysis revealed that cost of sac-TMT, progression-free survival (PFS) utility value, and discount rate were the three parameters exerting the strongest influence on the ICER. Probabilistic sensitivity analysis indicated that sac-TMT had a 23.90% probability of being cost-effective. Scenario analysis demonstrated that the ICER would precisely hit the cost-effectiveness threshold ($27,906.03/QALY) if the unit price of sac-TMT decreased to $541.55/200 mg.

**Conclusions:**

Based on this analytical model, sac-TMT is unlikely to be cost-effective for Chinese patients with EGFR-TKI–resistant, EGFR-mutated advanced NSCLC. Nevertheless, a further price reduction of 14.98% would make it cost-effective.

## Introduction

According to the International Agency for Research on Cancer, lung cancer was a major contributor to global cancer incidence and mortality in 2022, with 2.48 million new cases and 1.81 million deaths—accounting for 12.4% of all new cancer cases and 18.7% of all cancer deaths globally (1). Lung cancer ranks as the most prevalent malignancy in China, accounting for 22.0% of all newly diagnosed cancer cases and 28.5% of all cancer-related deaths (2). The disease is primarily classified into two pathological subtypes: non-small cell lung cancer (NSCLC) and small cell lung cancer (SCLC). NSCLC accounts for 80%−85% of all cases and represents the predominant clinical subtype. There are two major histological subtypes of NSCLC: non-squamous cell carcinoma and squamous cell carcinoma. Adenocarcinoma constitutes the most prevalent subtype. From the perspective of molecular classification, NSCLC can be categorized into driver gene-positive and driver gene-negative groups, with an approximate 1:1 ratio seen among patient populations (3). Patients with lung adenocarcinoma show high frequencies of actionable driver gene alterations, including targetable mutations in EGFR, ALK, ROS1, KRAS, BRAF, NTRK, MET exon 14 skipping alterations, and RET fusions. EGFR mutations are detected in 40%−50% of Chinese patients diagnosed with lung adenocarcinoma (4). Most patients with lung cancer are diagnosed with advanced-stage disease, which limits therapeutic alternatives and yields unfavorable overall prognoses (5). Epidermal growth factor receptor tyrosine kinase inhibitors (EGFR-TKIs) are effective for EGFR-sensitive NSCLC (6–8), but resistance typically develops within a year, limiting long-term benefits (9). Existing therapeutic strategies remain insufficient to meet clinical demands, highlighting an urgent need for novel antitumor agents to improve survival outcomes among patients with lung cancer (10–12).

The phase III OptiTROP-Lung 04 trial was a randomized, open-label, multicenter study conducted across 66 clinical sites in China. It enrolled patients with locally advanced or metastatic non-squamous NSCLC harboring EGFR mutations who had experienced disease progression following prior EGFR-TKI therapy and evaluated the efficacy and safety of sacituzumab tirumotecan (sac-TMT) vs. pemetrexed plus platinum-based chemotherapy. Superior survival outcomes were observed in the sac-TMT group, with a median progression-free survival (PFS) of 8.3 months, compared with 4.3 months in the chemotherapy arm (HR = 0.49; 95% CI: 0.39–0.62; *P* < 0.0001). The 18-month overall survival rate was also markedly higher with sac-TMT (65.8%) than with chemotherapy (48.0%) (HR = 0.60; 95% CI: 0.44–0.82; *P* = 0.001) (13).

The overall incidence of treatment-emergent adverse events (TEAEs) was similar between the two groups, at 58.0% for sac-TMT and 53.8% for pemetrexed–platinum chemotherapy. Neutropenia was the most reported adverse event, occurring in 39.9% of sac-TMT-treated patients and 33.0% of those receiving chemotherapy. Notably, patients administered sac-TMT had a substantially lower rate of treatment-related serious adverse events (9.0%) relative to the chemotherapy group (17.6%), confirming the favorable safety of sac-TMT (13).

In September 2025, the National Medical Products Administration (NMPA) of China granted approval to sac-TMT for adult patients with locally advanced or metastatic EGFR-mutated NSCLC whose disease had progressed following prior EGFR-TKI treatment. The OptiTROP-Lung04 trial, the first global clinical trial validating sac-TMT monotherapy efficacy after EGFR-TKI failure, serves as the pivotal clinical evidence supporting this regulatory approval. Trial data demonstrated that sac-TMT yields meaningful improvements in both PFS and OS for this patient population, conferring distinct clinical benefits when administered as a sequential regimen prior to platinum-based chemotherapy.

While sac-TMT shows significant clinical benefits for patients with locally advanced or metastatic non-squamous NSCLC after EGFR-TKI treatment progression, assessments of its cost-effectiveness in this group remain scarce. The existing pricing indicates that sac-TMT costs approximately $632.91 per 200 mg. Based on the recommended dosage of 5 mg/kg, a minimum of two doses is required for a single treatment, resulting in a cost that exceeds $2,547.98 per 28-day cycle. Furthermore, given that the drug must be administered biweekly, the long-term financial implications for patients are substantial and enduring. Therefore, it is crucial to analyze the cost-effectiveness of sac-TMT from the perspective of health systems in low-income and middle-income countries. This study aimed to evaluate the cost-effectiveness of sac-TMT for treating EGFR-mutated, locally advanced or metastatic non-squamous NSCLC that progressed after EGFR-TKI therapy, from the Chinese healthcare system's perspective. This evaluation aims to provide evidence to support rational drug use in clinical practice and inform medical decision-making by governmental bodies.

## Methods

This study is reported in accordance with the health economic evaluation requirements specified in the Consolidated Health Economic Evaluation Reporting Standards (CHEERS) 2022 Checklist (14). See Supplementary Table S5 for details.

### Patients and interventions

Eligible participants were adults (18–75 years) diagnosed with locally advanced (stage IIIB/C) or metastatic (stage IV) non-squamous NSCLC, confirmed via histology/cytology, for whom curative surgical resection or definitive chemoradiotherapy was not applicable. Tumor genotype was restricted to EGFR-sensitizing mutations, namely exon 19 deletions or the L858R substitution mutation in exon 21. Prior TKI therapy history dictated eligibility: having progressed on a first- or second-generation EGFR TKI and being T790M-negative, whereas progression following a third-generation TKI was sufficient regardless of T790M. All patients needed an Eastern Cooperative Oncology Group (ECOG) performance status of 0–1. Patients with brain metastases who had received treatment and were stable were permitted to participate (13).

sac-TMT was delivered intravenously at 5 mg/kg on days 1 and 15 of every 28-day cycle. The accompanying chemotherapy comprised pemetrexed (500 mg/m^2^) combined with either carboplatin (target AUC 5 mg/ml·min) or cisplatin (75 mg/m^2^), as selected by the investigator. Chemotherapy agents were administered on day 1 of each 21-day cycle for a maximum of four cycles, after which pemetrexed was continued as maintenance therapy. Treatment was maintained until disease progression, unacceptable toxicity, the patient's decision to withdraw, or the meeting of any other protocol-defined criteria for discontinuation.

The supplementary materials of sac-TMT indicated that after PD, the proportions of patients receiving at least one subsequent therapy were 85.50% and 72.30% in the two treatment groups, respectively (13). Given the wide variety of PD treatment regimens, many of which accounted for very low proportions and did not sum to 100%, this study selected the regimen with the largest proportion-namely, chemotherapy (41.9% vs. 53.6%)-as the treatment during PD (13). Referring to Guidelines of Chinese Society of Clinical Oncology (CSCO) for NSCLC, we adopted pemetrexed monotherapy as the treatment for the PD in this study (29). Patients who did not receive subsequent anticancer therapy were assumed to receive best supportive care.

### Data processing and evaluation

The data points from the PFS and overall survival (OS) curves were extracted from the literature using GetData Graph Digitizer software, and deviations among the data were documented. Deviations for all data points across the four survival curves predominantly fell within ± 0.05, indicating minimal data error and supporting the suitability of the data for reconstruction (Supplementary Figures S1–S4). The reconstructed hazard ratios (HR) for PFS and OS were 0.48 (95% CI: 0.38–0.61) and 0.59 (95% CI: 0.42–0.79), which show strong alignment with the original data. Furthermore, log cumulative hazard plots and smoothed hazard function plots were utilized to assess whether the survival functions of the two groups satisfied the proportional hazards (PH) assumption and exhibited a monotonic pattern, respectively (15). The log cumulative hazard plots (Supplementary Figures S5, S6) indicated that the PFS and OS curves for the chemotherapy and sac-TMT groups partially violated the PH assumption. The smoothed hazard function plots (Supplementary Figures S7, S8) revealed that the hazard functions of the four survival curves depart from linearity and monotonicity, suggesting a degree of inherent complexity. Schoenfeld residual plots (Supplementary Figures S9, S10) revealed a non-monotonic variation of the hazard ratios over time, further suggesting a potential violation of the proportional hazard assumption (16). The quantile-quantile plots (Supplementary Figures S11, S12) showed that the survival times of the two groups deviated from linearity with discernible inflection points, indicating that their survival distributions were not identical and suggesting a potential time-varying treatment effect.

Additionally, the OS data of the sac-TMT group and chemotherapy group also remained immature, with only 35.6% and 53.7% of events observed. All the aforementioned factors will significantly impact subsequent survival probability extrapolation (17–22).

Therefore, in addition to the standard parametric model (SPM, 8 sub-models, Supplementary Figures S13–S16), we incorporated the Royston-Parmar spline model (RPSM, 9 sub-models, Supplementary Figures S17–S20), the restricted cubic spline model (RCSM, 5 sub-models, Supplementary Figures S21–S24), the parameter mixture model (PMM, 28 sub-models, Supplementary Figures S25–S40), the fractional polynomial model (FPM, 44 sub-models, Supplementary Figures S41–S68), and the generalized additive model (GAM, 10 sub-models, Supplementary Figures S69–S72) to produce pseudo-individual time-to-event data and extrapolate survival probability according to the approach developed by Guyot et al. (23–27).

Given the substantial number of models considered in this study, a comprehensive comparison was conducted to evaluate their differences. Each model was assessed based on the mean squared error (MSE) of the median survival time against the original survival curve within the clinical trial follow-up period, the MSE of the survival probability at 12 months, and the MSEs of the restricted mean survival times (RMST) at 6, 12, and 18 months. The optimal model for survival rate extrapolation was subsequently selected by integrating the Akaike Information Criterion (AIC) with the models demonstrating relatively low cumulative MSE (Supplementary Tables S1–S4).

Ultimately, the optimal models for PFS and OS in the chemotherapy group were the 2-knot normal RPSM and the 1-knot normal RPSM, respectively. For the sac-TMT group, the optimal models for PFS and OS were the 3-knot odds RPSM and the 3-knot normal RPSM, respectively. The corresponding parameters for each optimal model are provided in Table 1. The extrapolated survival curves and the reconstructed survival curves are presented in Supplementary Figures S74–S77.

**Table 1:** Model parameters.

Parameters	Baseline	Range		Distribution	Source
Best model		Minimum	Maximum		
Progression-free survival curve of chemotherapy group in normal 2 knots					
gamma0	−1.93	−2.22	−1.65	MVN	Fitting
gamma1	0.85	0.40	1.29	MVN	Fitting
gamma2	−0.64	−1.25	−0.03	MVN	Fitting
gamma3	0.69	−0.01	1.39	MVN	Fitting
Progression-free survival curve of sac–TMT group in odds 3 knots					
gamma0	−3.61	−4.35	−2.88	MVN	Fitting
gamma1	2.47	1.33	3.61	MVN	Fitting
gamma2	0.55	−0.24	1.34	MVN	Fitting
gamma3	−0.57	−2.43	1.28	MVN	Fitting
gamma4	−0.04	−1.92	1.83	MVN	Fitting
Overall survival curve of chemotherapy group in normal 1 knot					
gamma0	−2.54	−3.06	−2.02	MVN	Fitting
gamma1	0.68	0.35	1.01	MVN	Fitting
gamma2	−0.13	−0.25	−0.01	MVN	Fitting
Overall survival curve of sac-TMT group in normal 3 knots					
gamma0	−2.64	−3.26	−2.02	MVN	Fitting
gamma1	0.59	−0.08	1.25	MVN	Fitting
gamma2	−0.21	−0.85	0.42	MVN	Fitting
gamma3	0.68	−1.57	2.93	MVN	Fitting
gamma4	−0.70	−3.28	1.88	MVN	Fitting
Cost					
sac-TMT (200 mg, 5 mg/kg)	637.00	509.60	764.40	Gamma	Local charge
Pemetrexed (100 mg, 500 mg/m^2^)	10.92	8.74	13.10	Gamma	Local charge
Carboplatin (30 mg, 75 mg/m^2^)	2.68	2.14	3.21	Gamma	Local charge
Per cycle (28 days)					
Pretreatment (sac-TMT group)	16.98	13.59	20.38	Gamma	Local charge
Pretreatment (chemotherapy group)	9.52	7.61	11.42	Gamma	Local charge
Laboratory tests and radiological examinations	397.70	318.16	477.24	Gamma	Local charge
Hospitalization	39.20	31.36	47.04	Gamma	Local charge
Terminal care	2,467.00	1,973.60	2,960.40	Gamma	(31)
Best supportive care	3,224.20	2,901.92	3,546.76	Gamma	(32)
Other (nursing, intravenous infusion, PICC catheterization, etc.)	55.04	44.35	66.05	Gamma	Local charge
Treatment for anemia	195.67	156.53	234.80	Gamma	Local charge
Treatment for leukopenia	702.81	562.25	843.37	Gamma	Local charge
Treatment for neutropenia	474.58	379.67	569.50	Gamma	Local charge
Treatment for thrombocytopenia	1,785.71	1,428.57	2,142.85	Gamma	Local charge
Utility					
PFS	0.740	0.680	0.800	Beta	(30)
PD	0.590	0.420	0.770	Beta	(30)
Anemia	−0.073	−0.066	−0.080	Beta	(33)
Leukopenia	−0.200	−0.180	−0.220	Beta	(31)
Neutropenia	−0.200	−0.180	−0.220	Beta	(31)
Thrombocytopenia	−0.190	−0.171	−0.209	Beta	(33)
Adverse events					
Intervention (%)					
Neutropenia	39.00%	31.20%	46.80%	Beta	(13)
Anemia	11.20%	8.96%	13.44%	Beta	(13)
Leukopenia	27.70%	22.16%	33.24%	Beta	(13)
Thrombocytopenia	2.10%	1.68%	2.52%	Beta	(13)
Control (%)					
Neutropenia	33.00%	26.40%	39.60%	Beta	(13)
Anemia	14.30%	11.44%	17.16%	Beta	(13)
Leukopenia	22.00%	17.60%	26.40%	Beta	(13)
Thrombocytopenia	16.50%	13.20%	19.80%	Beta	(13)
**Body weight**	65	52	78	Gamma	(34)
**Body surface area (m** ^**2**^ **)**	1.680	1.344	2.016	Gamma	(34)
**Discount rate**	0.045	0	0.05	Fixed	(28)
**Glomerular filtration rate (ml/min)**	70	56	84	Gamma	(35)

MVN, multivariate normal distribution; sac-TMT, sacituzumab tirumotecan; PFS, Progression-free survival; PD, Progressed disease.

### Model structure

To estimate the distribution of patients across health states over time, we developed a 10-year partitioned survival model—a standard methodology in oncology economic evaluations. As shown in Supplementary Figure S73, the model consists of three mutually exclusive states: PFS, progressed disease (PD), and death. The proportion of patients in the PFS state at a given time *t* is derived directly from the PFS curve, denoted as PFS(t). The OS curve indicates the proportion of patients who are still alive at time t, denoted as OS(t). From these, the proportion in the PD state is calculated as OS(t)—PFS(t), while the proportion who have died is given by 1 – OS(t). To reconstruct individual patient time-to-event data and conduct survival extrapolation, we utilized specialized R packages, namely “flexsurv”, “survHE”, and “IPDfromKM”. The model framework was then operationalized in Microsoft Excel 365, thereby ensuring full transparency and enabling the step-by-step visualization of all computations.

We used a 28-day cycle for the sac-TMT group and the chemotherapy group. Following contemporary modeling conventions, half-cycle correction was applied in this study to properly address the transition between cycles. The threshold for willingness to pay (WTP) was set at $27,906.03/QALY, which is 2 times the Gross Domestic Product (GDP) per person in 2025 (28). In accordance with the Chinese Pharmacoeconomic Evaluation Guidelines (version 2025), we applied a 4.5% annual discount rate to both costs and outcomes, with all costs adjusted according to the Consumer Price Index (CPI) (28). Currency conversion utilized the 2025 average exchange rate of 1 CNY = 0.139 USD. The study evaluated outcomes based on total costs, quality-adjusted life years (QALYs), the incremental cost-effectiveness ratio (ICER), and life-years gained (LYs).

### Costs and utilities

From a healthcare system perspective, direct medical costs were considered, including drug expenses, diagnostic tests, hospitalization fees, and treatment costs for adverse events (AEs). As stated in the Patients and Interventions section, the cost of sac-TMT was $2,547.98 per 28-day cycle, while that of chemotherapy stood at $202.70 for each 28-day cycle. Only AEs demonstrated 5% frequency and severe (grade ≥3) manifestations (e.g., leukopenia, anemia, thrombocytopenia, neutropenia) were included. The corresponding cost data were obtained through expert consultation conducted at local hospitals, whereas the disutility were extracted from two pharmacoeconomic studies (31, 33). The utility for the PFS and PD phases in second-line treatment of advanced NSCLC were 0.74 QALYs and 0.59 QALYs, respectively (30). Cost for terminal care constituted a non-negligible component among patients with advanced NSCLC. Given substantial variations driven by individual, familial and social factors, we adopted the value of $2467 from previously published literature (31). We assumed that patients who did not receive subsequent antitumor therapies would receive best supportive care, with daily cost parameters derived from the study by Li et al. ($115.15 per day) (32). The corresponding 28-day cost amounted to $3,224.20, which was incorporated into the model as a one-time expense. Likewise, AEs were assumed to rise exclusively within the first treatment cycle, with corresponding treatment costs only accrued during this initial cycle. Physiological parameters including body weight, body surface area and glomerular filtration rate (GFR) were set at 65 kg, 1.68 m^2^ and 70 ml/min, respectively, with data retrieved from published studies (34, 35). The annual discount rate of 4.50% was converted to match the 28-day cycle (28). All cost and utility parameters, along with their respective sources, are detailed in Table 1.

### Sensitivity analysis

One-way sensitivity analysis (OWSA) was conducted to examine the impact of varying parameters on the ICER, with results presented in a tornado diagram. Probabilistic sensitivity analysis (PSA) was conducted by assigning normal, gamma, and beta distributions to model parameters and running 1,000 Monte Carlo simulations. The results are presented in scatter plots to assess model stability. Given the immaturity of OS data in the sac-TMT group, we employed the repeated one-way sensitivity analyses (ROWSA) to evaluate the impact of extrapolated survival probability, randomly varied within the confidence interval according to a normal distribution, on ICER. The extrapolated survival data were subjected to 1,000 bootstrap iterations within their respective confidence intervals (Supplementary Figures S78–S81). The area under the curve (AUC) was then calculated using the first-order Newton-Cotes closed integration formula, followed by frequency distribution analysis for AUCs of 1,000 survival curves (Supplementary Figures S82–S85). In the probabilistic sensitivity analysis (PSA), survival curve uncertainty was considered, and the outcomes were presented as scatter plots and cost-effectiveness acceptability curve (CEAC) after 1,000 Monte Carlo simulations.

### Scenario analysis

Considering the comparatively elevated expense of sac-TMT, we decreased its price in 10% increments until achieving a 90% reduction from the initial price, calculating the associated ICER at each interval. Similarly, we also conducted a scenario analysis on the time horizon, starting from 1 year and increasing annually up to 10 years.

### Subgroup analysis

Based on the HRs for PFS and overall survival OS pooled from clinical trials across different subgroups, we calculated the ICER for each subgroup by applying these HRs to the baseline outcome data.

## Results

### Base-case analysis

Table 2 presented the base analysis of the 10-year PSM. Compared with chemotherapy group ($29,006.04; 1.01 QALYs; 1.70 LYs), sac-TMT group demonstrated higher total costs, effectiveness and LYs ($57,291.32; 1.85 QALYs; 3.43 LYs). The incremental cost and incremental effectiveness were $28,285.28 and 0.84 QALYs, respectively, yielding an ICER of $33,514.79/QALY. It exceeded the WTP threshold of $27,906.03/QALY set in this study.

**Table 2:** Results of base-case analysis.

Strategy	Cost ($)	QALYs	Incremental cost ($)	Incremental QALYs	ICER ($/QALY)	LYs
Sacituzumab tirumotecan	57,291.32	1.85	28,285.28	0.84	33,514.79	3.43
Chemotherapy	29,006.04	1.01				1.70

QALY, quality-adjusted life year; ICER, incremental cost-effectiveness ratio.

### Scenario analysis

As shown in Table 3, the scenario analysis demonstrated that reducing the price of sac-TMT lowered the corresponding ICER. When the drug price was reduced by 20% ($509.60/200 mg), the resulting ICER of $26,028.16/QALY fell below the cost-effectiveness threshold. When the price was reduced by 14.98% to $541.55/200 mg, the ICER reached the threshold.

**Table 3:** Results of scenario analysis (price reduction).

Price reduction	ICER ($/QALY)
10%	29,771.46
20%	26,028.16
30%	22,284.79
40%	18,541.46
50%	14,798.13
60%	11,054.79
70%	7,311.46
80%	3,568.13
90%	^*^

“^*^”, Sacituzumab tirumotecan is dominant.

In the time horizon scenario analysis (Table 4), sac-TMT remained not cost-effective compared to chemotherapy when the time horizon was extended from 1 year to 10 years. As the time horizon increases, the ICER gradually declines, but the reductions became smaller in magnitude. At year 9, the ICER was $34,181.73/QALY, which differs by $666.94/QALY from the ICER in this study at a 10-year horizon.

**Table 4:** Results of scenario analysis (time horizon).

Time horizon (year)	Incremental cost ($)	Incremental QALYs	Incremental LYs	ICER ($/QALY)
1	15,035.74	0.05	0.05	292,729.97
2	17,359.43	0.16	0.22	108,874.46
3	18,384.95	0.26	0.41	71,258.95
4	21,588.17	0.45	0.78	48,459.38
5	23,459.02	0.56	1.02	41,964.66
6	24,949.35	0.65	1.23	38,507.37
7	26,018.07	0.71	1.38	36,533.85
8	26,959.65	0.77	1.52	35,143.61
9	27,691.07	0.81	1.64	34,181.73
10	28,285.28	0.84	1.74	33,514.79

### Subgroup analysis

Under the proportional hazards assumption, subgroup analyses summarized in Table 5 demonstrated that sac-TMT failed to show cost-effective as a therapeutic strategy for EGFR-TKI–resistant, EGFR-mutated advanced NSCLC, across all patient subgroups. The subgroup with the lowest ICER was patient with brain metastases, with an ICER of $32,145.42/QALY. By contrast, the subgroup comprising patients with a baseline ECOG performance status score of 0 yielded the highest ICER, valued at $49,396.83/QALY.

**Table 5:** Results of subgroup analysis.

Subgroup	HR of PFS	HR of OS	ICER ($/QALY)
Sex			
Male	0.53	0.7	44,084.09
Female	0.44	0.54	43,403.82
Age			
<65 years	0.46	0.55	42,441.71
≥65 years	0.51	0.62	42.018.54
History of smoking			
Current or former smoker	0.56	0.71	42,583.83
Never smoked	0.44	0.55	43,825.79
ECOG performance-status score			
0	0.38	0.57	49,396.83
1	0.5	0.59	41,444.43
Previous third-generation EGFR-TKI therapy			
First-line therapy	0.48	0.59	42,746.47
Second-line therapy	0.49	0.58	41,682.51
Brain metastases			
Yes	0.73	0.65	32,145.42
No	0.43	0.57	45,419.93
Liver metastases			
Yes	0.54	0.53	36,884.08
No	0.46	0.6	44,549.95
EGFR mutation subtype			
Exon 21 L858R substitution	0.57	0.75	43.662.31
Exon 19 deletion	0.42	0.46	41,520.62
T790M mutation status			
Negative	0.5	0.67	44,803.67
Positive	0.5	0.36	33,422.12
Unknown	0.45	0.62	46,164.90

### Sensitivity analysis

**Figure 1:**
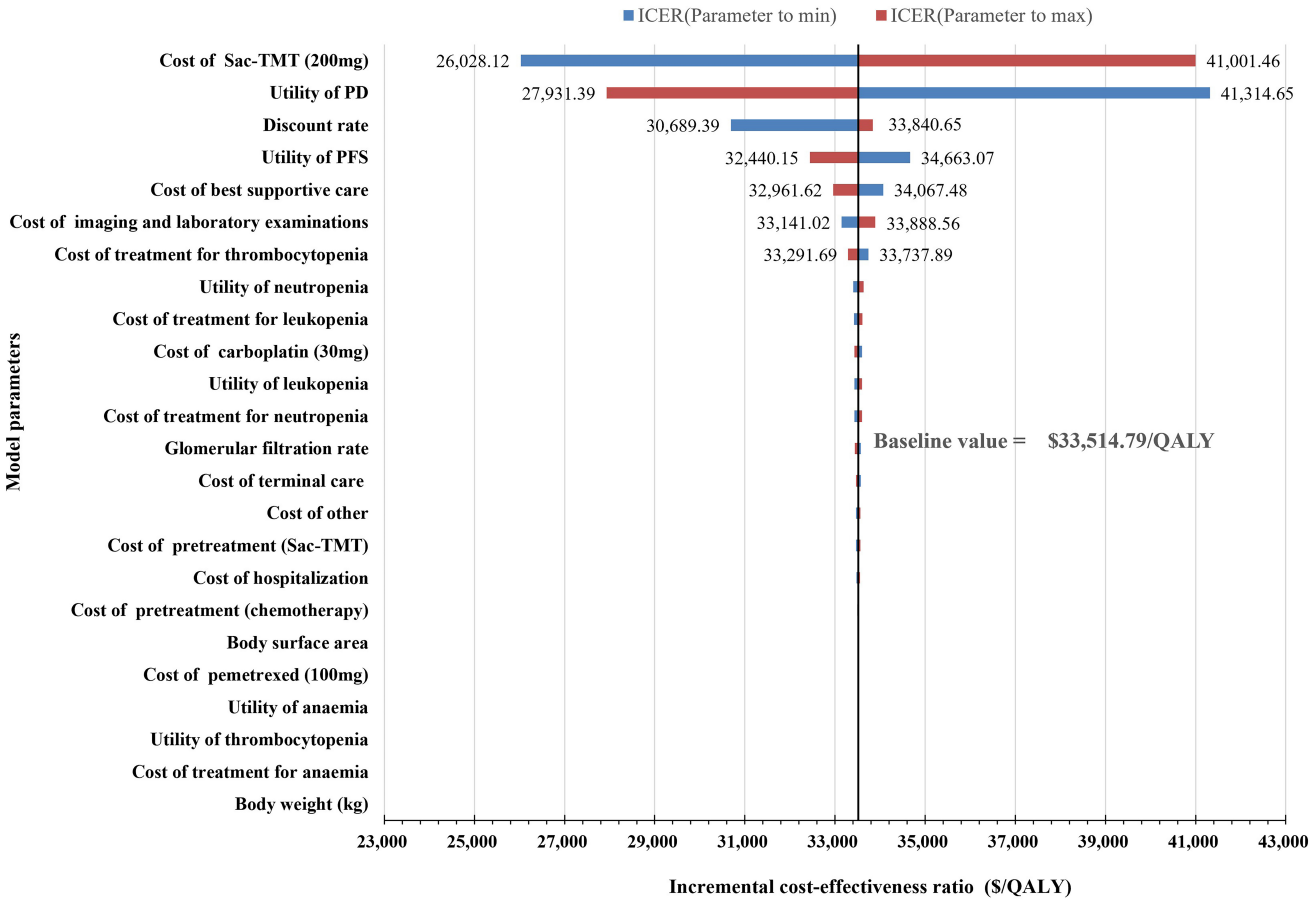
The tornado diagram of deterministic sensitivity analysis.

**Figure 2:**
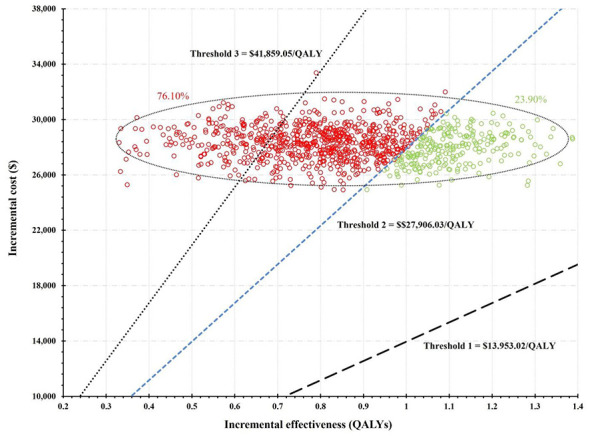
Scatter plot (1,000 Monte Carlo process).

**Figure 3:**
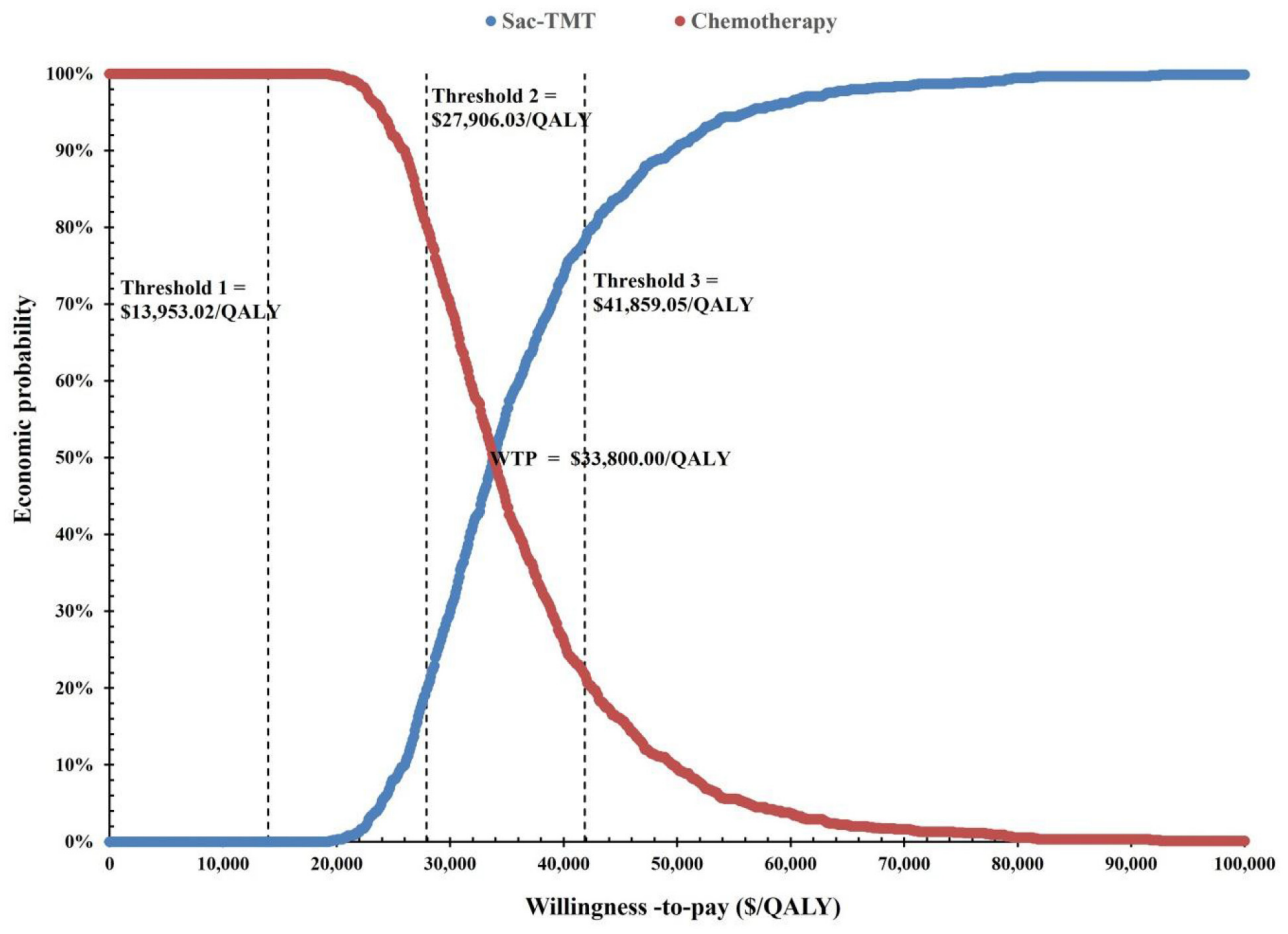
The cost-effectiveness acceptability curve.

**Figure 4:**
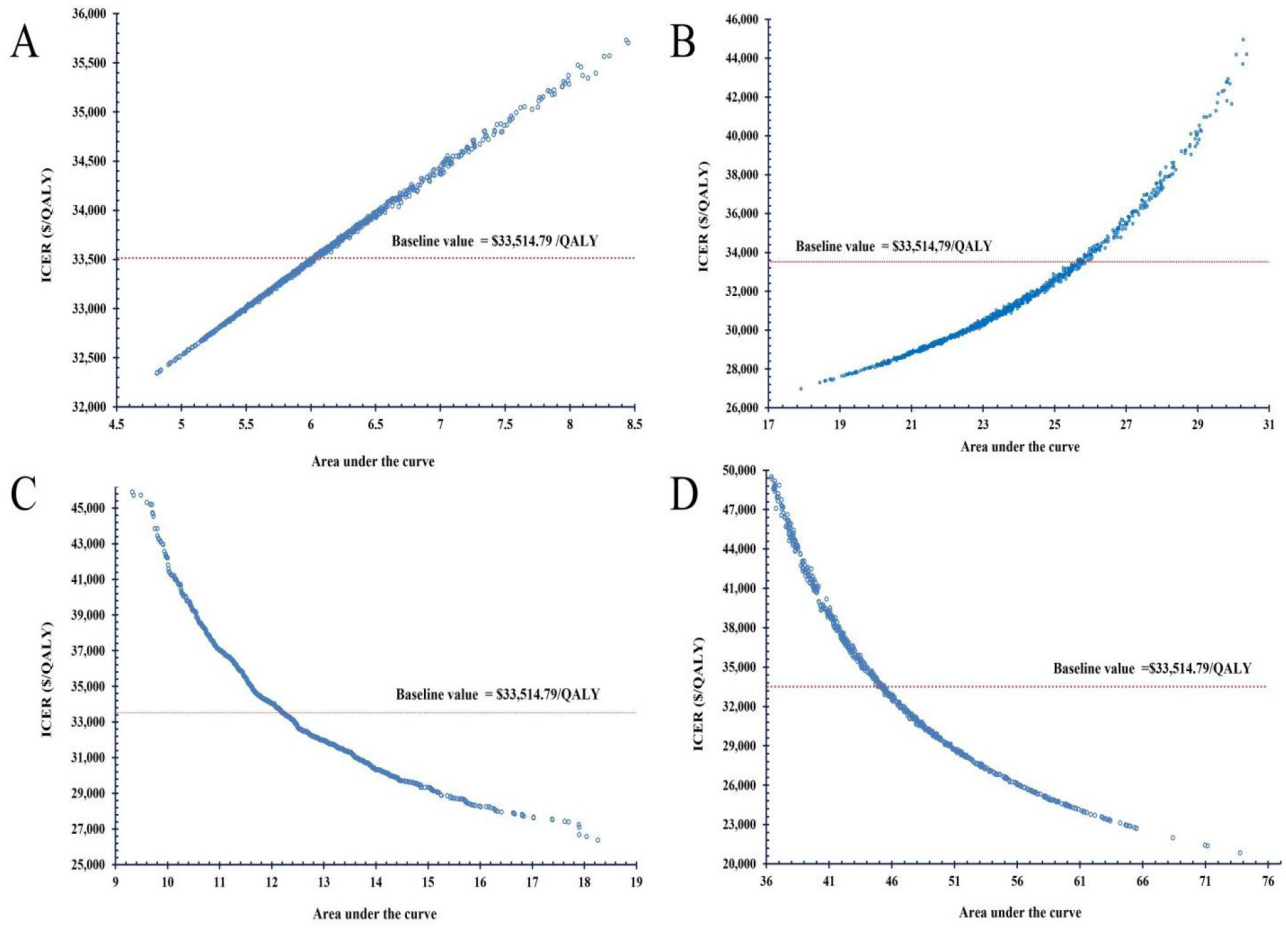
Repeated one-way sensitivity analysis (1,000 Monte Carlo process). **(A)** Progressive-free survival of chemotherapy group; **(B)** Overall survival of chemotherapy group; **(C)** Progressive-free survival of sacituzumab tirumotecan group; **(D)** Overall survival of sacituzumab tirumotecan group.

In the OWSA (Figure 1), varying the cost parameter by ±20% and the remaining parameters across their reported ranges showed that the three factors most influential on the ICER were the cost of sac-TMT, the utility of PD, and the discount rate. Other parameters had minimal impact. The results of the PSA (Figure 2) indicated that when model parameters were varied randomly according to their specified distributions, the proportions of ICER points above the first threshold (1 time GDP per capita), the second threshold (2 times GDP per capita), and the third threshold (3 times GDP per capita) were 100.00%, 76.10%, and 19.30%, respectively. The CEAC (Figure 3) demonstrated that at a WTP threshold of $33,800.00/QALY, chemotherapy and Sac-TMT had the same probability of being cost-effective. The ROWSA (Figure 4) demonstrated that for the sac-TMT group, as the AUC of PFS and OS increased, the ICER progressively decreased. Conversely, the opposite trend was observed in the chemotherapy group. Due to uncertainties in survival curve extrapolation, variations in extrapolated survival probability resulted in ICER fluctuations across the following ranges ($/QALY): 32,342.10–35,703.59 (Figure 4A), 26,969.05–44,946.59 (Figure 4B), 26,374.20–45,899.10 (Figure 4C), and 20,143.38–49,488.70 (Figure 4D). Notably, ICER values fell below the threshold when the AUC of extrapolated OS curves for the chemotherapy group ranged from 17.92 to 19.51 (proportion: 21/1,000). For the sac-TMT group, ICERs were under the threshold when the AUCs of extrapolated PFS and OS curves fell within the intervals of 16.64–18.26 (proportion: 18/1,000) and 52.50–77.88 (proportion: 134/1,000), respectively.

## Discussion

Within this study's analytical framework, sac-TMT compared to chemotherapy alone yields an ICER of $33,514.79/QALY, exceeding the predefined cost-effectiveness threshold. This suggests the sac-TMT, as a treatment for EGFR-TKI–resistant, EGFR-mutated advanced NSCLC patients, may not be cost-effective in China. Sensitivity analyses verified the robustness of these findings. The vast majority of resultant ICER values exceeded the threshold, regardless of whether parameters were varied or extrapolated survival curves fluctuated within their corresponding confidence intervals. The immaturity of OS data in the sac-TMT group introduced substantial uncertainty in survival curve extrapolation, resulting in the widest ICER variation range.

According to the China Statistical Yearbook 2025, the three provincial-level regions with the highest GDP per capita are Beijing ($33,177.99), Shanghai ($31,803.82), and Jiangsu ($23,224.41) (36). The corresponding two-times GDP thresholds are $66,355.99/QALY, $63,607.65/QALY, and $46,448.83/QALY, respectively. Thus, in the context of China's most economically developed regions, sac-TMT is cost-effective compared with chemotherapy. This regional heterogeneity in cost-effectiveness aligns with the institutional reality of provincial-level medical insurance pooling in China. Economically developed provinces often supplement the national reimbursement drug list (NRDL) with local supplementary insurance and inclusive commercial health insurance programs, which can afford higher-value innovative therapies and achieve optimal local health resource allocation (37, 38).

Numerous economic evaluations have been conducted on second-line therapies for advanced NSCLC. Osimertinib ($1,680.00/21days) yielded an ICER of $25,821.00/QALY compared to chemotherapy for EGFR-mutated advanced NSCLC (31). Compared to placebo group, nivolumab group ($2,786.88/14days) resulted in incremental costs of $72,127.71 for platinum-treated advanced NSCLC (39). The base analysis showed trastuzumab deruxtecan group ($9,716.00/days) compared to docetaxel generated an ICER of $137,959.45/QALY for HER2-mutant advanced NSCLC (40). Sotorasib group ($16,892.82/21days) compared to chemotherapy group, it demonstrated an ICER of $102,701.84/QALY for KRAS^G12C^-mutant advanced NSCLC (41). Lijun Jin et al. found that sac-TMT ($5,263.40/28days) yielded an ICER of $87,922.33/QALY compared with chemotherapy for EGFR-TKI–resistant, EGFR-mutated advanced NSCLC (42). In these studies, only osimertinib indicates cost-effectiveness, driven largely by its oral administration route, lower delivery costs, and substantial price reductions through successive national price negotiations. The unfavorable cost-effectiveness of immune checkpoint inhibitors such as nivolumab is attributed to their high price. For antibody-drug conjugates (ADCs) such as trastuzumab deruxtecan, sac-TMT exorbitant drug acquisition costs remain the primary barrier to cost-effectiveness. Consistent with our expectations, sac-TMT does not demonstrate cost-effectiveness, which further bolsters the reliability of our analytical results.

In China, the total economic burden of lung cancer reaches approximately $25,069 billion in 2017, accounting for 0.121% of GDP that year (43). By 2019, lung cancer resulted in 832.27 disability-adjusted life years (DALYs) per 100,000 population (44). Recent study estimated that the adoption of PD-1/PD-L1 inhibitors in China from 2023 to 2027 will generate an additional 247,374 QALYs and prolong PFS by 226,374 years among patients with NSCLC (45). Although the initial market prices of these drugs are prohibitively high, substantially limiting their clinical utilization, the National Medication Price Negotiation Policy (NMPNP) has dramatically improved both the affordability and availability of innovative pharmaceuticals (46). The 2025 NRDL negotiation concludes with 114 newly added medicines, including 36 cancer drugs, achieving an average price reduction of 63% (47). Sac-TMT also successfully passed the negotiation, with its price reduced from $1,315.85/200 mg to $637.00/200 mg. But it only demonstrates cost-effectiveness when its cost reduces by 14.98% again. This indicates that sac-TMT would require a more substantial price reduction in future national reimbursement negotiations to demonstrate cost-effectiveness.

In pharmacoeconomic evaluations, uncertainty is systematically categorized by source into four distinct types: structural uncertainty, between-patient heterogeneity, parameter uncertainty, and stochastic uncertainty (48). This study is based on the OptiTROP-Lung 04 trial, where baseline characteristics showed no statistically significant differences between the two treatment arms (13). However, due to the unavailability of individual patient-level data (IPD), we are unable to assess the stochastic variability in per-patient cost and effectiveness (49). Furthermore, structural uncertainty, which is inherently tied to model assumptions, is difficult to capture through conventional sensitivity analyses (50, 51). Given the immature OS data for sac-TMT, substantial uncertainty exists in extrapolating the OS curve even when using optimal fitting models. To address the complex hazard function and local n-PH observed across four survival curves, we employ six classes of extrapolation models, each further subdivided into 104 sub-models by mathematical methodology or curve characteristics (Supplementary Tables S1–S4). To select the optimal extrapolation model, we evaluate the goodness-of-fit based on the MSE across three key metrics: survival probabilities, RMST at sequential time points, and median survival time—all compared between the extrapolated and original curves during the follow-up. This multi-metric MSE-based method, rather than dependence on AIC or BIC alone, is intended to facilitate the selection of a model that more accurately reflects the true survival trajectory. Finally, we employ an RPSM for survival extrapolation, which provides greater flexibility than standard parametric models in addressing both analytical challenges (21). Limited follow-up duration (max: 24 months) in the OS dataset of the sac-TMT group prevented correction of extrapolated survival probability exceeding 2-year horizons. Therefore, we conduct ROWSA on the four extrapolated survival datasets, which can observe the range of influence on ICER. As expected, the uncertainty surrounding OS data produced the greatest fluctuation in ICER, yielding a range of $29,345.32/QALY. As shown in Figure 4, in the order presented, the four survival curves had 0%, 2.1%, 1.8%, and 13.4% of their ICER values below the threshold, respectively. Notably, the vast majority of ICERs remained above the threshold, thereby reinforcing the robustness of our findings.

This study has several limitations. First, the treatment options after PD were oversimplified, as subsequent-line therapies (e.g., targeted therapies, immunotherapy, and radiotherapy) were not proportionally incorporated. However, OWSA demonstrated the minimal impact of PD-phase treatment costs on ICERs. Second, regarding parameter variation, we chose a rather crude range of ± 20% on cost, which might not fully capture the uncertainty surrounding the ICER. Fourth, although we fitted several models, we did not examine ICERs derived from models other than the RPSM. However, compared with the SPM model selected by Lijun Jin et al., the ICER value is unlikely to fall below the threshold (42). Fifth, the immaturity of the overall survival data and the absence of utility values from the OptiTROP-Lung 04 trial inevitably introduced bias into the results. Sixth, the costs of best supportive care and terminal care were not adjusted to the 2025 US dollar exchange rate. Nevertheless, DSA results indicated that these parameters exerted only a minor influence on the ICER. Finally, all clinical efficacy data are derived from the OptiTROP-Lung 04 trial, which might differ from real-world outcomes—a discrepancy requiring future investigation.

## Conclusion

Based on a 10-year PSM, sac-TMT is unlikely to be a cost-effective therapy compared with chemotherapy for patients in China with EGFR-TKI–resistant, EGFR-mutated advanced NSCLC. Nevertheless, if sac-TMT attains a 14.98% price reduction again, it would likely achieve cost-effectiveness.

## Data Availability

The original contributions presented in the study are included in the article/Supplementary material, further inquiries can be directed to the corresponding authors.
