# Supplementary material for: Cost-effectiveness analysis of sacituzumab tirumotecan vs. chemotherapy for patients with EGFR-TKI–resistant, EGFR-mutated advanced non-small cell lung cancer in China
Source: Front Public Health. 2026 Jul 27;14:1843636. doi: 10.3389/fpubh.2026.1843636 (PMC13453752; doi:10.3389/fpubh.2026.1843636)
Supplement: Supplementary file 1 [file Supplementary_file_1.docx]

**Cost-effectiveness analysis of sacituzumab tirumotecan versus chemotherapy for patients with EGFR-TKI–resistant, EGFR-mutated advanced non-small cell lung cancer in China**

Kai Xu^1^, Yuting Yan^2^, Zhaoliu Cao^3^, Jinchun Liu^4^, Hongting Yao^5^, Yuyang sun^5^, Jingyu Zhang^6^*, Hong Wu^7^*

^1^ Pharmacy Department, Nanjing City QiXia District Qixia community healthcare center, No. 47 QiXia South Street, Nanjing, Jiangsu, People’s Republic of China.

^2^ Songjiang Research Institute, Shanghai Key Laboratory of Emotions and Affective Disorders, Songjiang District Central Hospital, Shanghai Jiao Tong University School of Medicine, Shanghai, People’s Republic of China.

^3^ Pharmacy Department, Nanjing City QiXia District Hospital, No. 28 Yaojia Road, Qixia District, Nanjing City, Jiangsu Province, People’s Republic of China.

^4^ Department of Pharmacy, Nanjing Drum Tower Hospital, No.321 Zhongshan Road, Gulou District, Naniing City, Jiangsu Province, People's Republic of China.

^5^ Department of Pharmaceutical Regulatory Science and Pharmacoeconomics, School of Pharmacy, Nanjing Medical University, Nanjing, Jiangsu, People’s Republic of China.

^6^ Department of Oncology, Affiliated Lianyungang Clinical College of Nantong University, Lianyungang 222000, Jiangsu, People’s Republic of China.

^7^ Department of Pharmacy, The Second People's Hospital of Lianyungang,No. 41 Hailian East Road, Haizhou District Lianyungang, Jiangsu,222000, People’s Republic of China.

Correspondence: Hong Wu, Department of Pharmacy, The Second People's Hospital of Lianyungang,No. 41 Hailian East Road, Haizhou District Lianyungang, Jiangsu,222000, People’s Republic of China; Jingyu Zhang, Department of Oncology, Affiliated Lianyungang Clinical College of Nantong University, Lianyungang 222000, Jiangsu, People’s Republic of China.

**Directory**

[1. Data quality evaluation	- 1 -](#_Toc22841)

[2. Assessment of the proportional hazards assumption	- 5 -](#_Toc8546)

[3. Test of hazard function complexity	- 7 -](#_Toc713)

[4. Schoenfeld test and Wald test	- 9 -](#_Toc7900)

[5. Quantile-Quantile plot	- 11 -](#_Toc32764)

[6. Fitting in standard parametric model	- 13 -](#_Toc6658)

[7. Fitting in RP spline model	- 17 -](#_Toc15213)

[8. Fitting in restricted cubic spline model	- 21 -](#_Toc20901)

[9. Fitting in parameter mixture model	- 25 -](#_Toc1220)

[10. Fitting in fractional polynomial model	- 41 -](#_Toc6779)

[11. Fitting in generalized additive model	- 69 -](#_Toc23243)

[12. The selection of best model	- 73 -](#_Toc10608)

[13. Model structure	- 93 -](#_Toc4854)

[14. The best model	- 94 -](#_Toc2417)

[15. The reconstructed curve	- 96 -](#_Toc12935)

[16. Distrubution of bootstrap sampling for survival curve	- 98 -](#_Toc24737)

[17. The reuslts of 1,000 bootstrap sampling for survival curve	- 102 -](#_Toc11634)

[18. CHEERS 2022 Checklist	- 106 -](#_Toc2068)

# 1. Data quality evaluation

Figure S1. The data quality evaluation of progression-free survival curve in chemotherapy group

Figure S2. The data quality evaluation of overall survival curve in chemotherapy group

Figure S3. The data quality evaluation of progression-free survival curve in sacituzumab tirumotecan group

Figure S4. The data quality evaluation of overall survival curve in sacituzumab tirumotecan group

# 2. Assessment of the proportional hazards assumption

Figure S5. The assessment of the proportional hazards assumption for progression-free survival curve

Figure S6. The assessment of the proportional hazards assumption for overall survival curve

# 3. Test of hazard function complexity

Figure S7. The test of hazard function complexity for progression-free survival curve

Figure S8. The test of hazard function complexity for overall survival curve

# 4. Schoenfeld test and Wald test

Figure S9. The Schoenfeld test and Wald test for progression-free survival curve

Figure S10. The Schoenfeld test and Wald test for overall survival curve

# 5. Quantile-Quantile plot

Figure S11. The Q-Q plot for progression-free survival curve

Figure S12. The Q-Q plot for overall survival curve

# 6. Fitting in standard parametric model

Figure S13. Fitting of progression-free survival curve for chemotherapy group in standard parametric model

Figure S14. Fitting of overall survival curve for chemotherapy group in standard parametric model

Figure S15. Fitting of progression-free survival curve for sacituzumab tirumotecan group in standard parametric model

Figure S16. Fitting of overall survival curve for sacituzumab tirumotecan group in standard parametric model

# 7. Fitting in RP spline model

Figure S17. Fitting of progression-free survival curve for chemotherapy group in RP spline model

Figure S18. Fitting of overall survival curve for chemotherapy group in RP spline model

Figure S19. Fitting of progression-free survival curve for sacituzumab tirumotecan group in RP spline model

Figure S20. Fitting of overall survival curve for sacituzumab tirumotecan group in RP spline model

# 8. Fitting in restricted cubic spline model

Figure S21. Fitting of progression-free survival curve for chemotherapy group in restricted cubic spline model

Figure S22. Fitting of overall survival curve for chemotherapy group in restricted cubic spline model

Figure S23. Fitting of progression-free survival curve for sacituzumab tirumotecan group in restricted cubic spline model

Figure S24. Fitting of overall survival curve for sacituzumab tirumotecan group in restricted cubic spline model

# 9. Fitting in parameter mixture model

Figure S25. Fitting of progression-free survival curve for chemotherapy group in parameter mixture model (model 1-7)

Figure S26. Fitting of progression-free survival curve for chemotherapy group in parameter mixture model (model 8-14)

Figure S27. Fitting of progression-free survival curve for chemotherapy group in parameter mixture model (model 15-21)

Figure S28. Fitting of progression-free survival curve for chemotherapy group in parameter mixture model (model 22-28)

Figure S29. Fitting of overall survival curve for chemotherapy group in parameter mixture model (model 1-7)

Figure S30. Fitting of overall survival curve for chemotherapy group in parameter mixture model (model 8-14)

Figure S31. Fitting of overall survival curve for chemotherapy group in parameter mixture model (model 15-21)

Figure S32. Fitting of overall survival curve for chemotherapy group in parameter mixture model (model 22-28)

Figure S33. Fitting of progression-free survival curve for sacituzumab tirumotecan group in parameter mixture model (model 1-7)

Figure S34. Fitting of progression-free survival curve for sacituzumab tirumotecan group in parameter mixture model (model 8-14)

Figure S35. Fitting of progression-free survival curve for sacituzumab tirumotecan group in parameter mixture model (model 15-21)

Figure S36. Fitting of progression-free survival curve for sacituzumab tirumotecan group in parameter mixture model (model 22-28)

Figure S37. Fitting of overall survival curve for sacituzumab tirumotecan group in parameter mixture model (model 1-7)

Figure S38. Fitting of overall survival curve for sacituzumab tirumotecan group in parameter mixture model (model 8-14)

Figure S39. Fitting of overall survival curve for sacituzumab tirumotecan group in parameter mixture model (model 15-21)

Figure S40. Fitting of overall survival curve for sacituzumab tirumotecan group in parameter mixture model (model 22-28)

# 10. Fitting in fractional polynomial model

Figure S41. Fitting of progression-free survival curve for chemotherapy group in first-order fractional polynomial model

Figure S42. Fitting of progression-free survival curve for chemotherapy group in second-order fractional polynomial model (model 1-6)

Figure S43. Fitting of progression-free survival curve for chemotherapy group in second-order fractional polynomial model (model 7-12)

Figure S44. Fitting of progression-free survival curve for chemotherapy group in second-order fractional polynomial model (model 13-18)

Figure S45. Fitting of progression-free survival curve for chemotherapy group in second-order fractional polynomial model (model 19-24)

Figure S46. Fitting of progression-free survival curve for chemotherapy group in second-order fractional polynomial model (model 25-30)

Figure S47. Fitting of progression-free survival curve for chemotherapy group in second-order fractional polynomial model (model 31-36)

Figure S48. Fitting of overall survival curve for chemotherapy group in first-order fractional polynomial model

Figure S49. Fitting of overall survival curve for chemotherapy group in second-order fractional polynomial model (model 1-6)

Figure S50. Fitting of overall survival curve for chemotherapy group in second-order fractional polynomial model (model 7-12)

Figure S51. Fitting of overall survival curve for chemotherapy group in second-order fractional polynomial model (model 13-18)

Figure S52. Fitting of overall survival curve for chemotherapy group in second-order fractional polynomial model (model 19-24)

Figure S53. Fitting of overall survival curve for chemotherapy group in second-order fractional polynomial model (model 25-30)

Figure S54. Fitting of overall survival curve for chemotherapy group in second-order fractional polynomial model (model 31-36)

Figure S55. Fitting of progression-free survival curve for sacituzumab tirumotecan group in first-order fractional polynomial model

Figure S56. Fitting of progression-free survival curve for sacituzumab tirumotecan group in second-order fractional polynomial model (model 1-6)

Figure S57. Fitting of progression-free survival curve for sacituzumab tirumotecan group in second-order fractional polynomial model (model 7-12)

Figure S58. Fitting of progression-free survival curve for sacituzumab tirumotecan group in second-order fractional polynomial model (model 13-18)

Figure S59. Fitting of progression-free survival curve for sacituzumab tirumotecan group in second-order fractional polynomial model (model 19-24)

Figure S60. Fitting of progression-free survival curve for sacituzumab tirumotecan group in second-order fractional polynomial model (model 25-30)

Figure S61. Fitting of progression-free survival curve for sacituzumab tirumotecan group in second-order fractional polynomial model (model 31-36)

Figure S62. Fitting of overall survival curve for sacituzumab tirumotecan group in first-order fractional polynomial model

Figure S63. Fitting of overall survival curve for sacituzumab tirumotecan group in second-order fractional polynomial model (model 1-6)

Figure S64. Fitting of overall survival curve for sacituzumab tirumotecan group in second-order fractional polynomial model (model 7-12)

Figure S65. Fitting of overall survival curve for sacituzumab tirumotecan group in second-order fractional polynomial model (model 13-18)

Figure S66. Fitting of overall survival curve for sacituzumab tirumotecan group in second-order fractional polynomial model (model 19-24)

Figure S67. Fitting of overall survival curve for sacituzumab tirumotecan group in second-order fractional polynomial model (model 25-30)

Figure S68. Fitting of overall survival curve for sacituzumab tirumotecan group in second-order fractional polynomial model (model 31-36)

# 11. Fitting in generalized additive model

Figure S69. Fitting of progression-free survival curve for chemotherapy group in generalized additive model

Figure S70. Fitting of overall survival curve for chemotherapy group in generalized additive model

Figure S71. Fitting of progression-free survival curve for sacituzumab tirumotecan group in generalized additive model

Figure S72. Fitting of overall survival curve for sacituzumab tirumotecan group in generalized additive model

# 12. The selection of best model

Table S1. The selection of best model for progression-free survival curve in chemotherapy group

|  | ***AIC*** | ***Log Likelihood*** | ***MSE of median PFS*** | ***MSE of 12-m SP*** | ***MSE of 6-m RMST*** | ***MSE of 12-m RMST*** | ***MSE of 18-m RMST*** | ***Cumulative MSE*** |
| --- | --- | --- | --- | --- | --- | --- | --- | --- |
| ***Standard parametric model*** |  |  |  |  |  |  |  |  |
| Exponential | 900.99 | -449.49 | 0.03430 | 0.00310 | 0.26920 | 0.01070 | 0.00220 | 0.31950 |
| Weibull | 866.36 | -431.18 | 0.39950 | 0.00000 | 0.00010 | 0.06030 | 0.00700 | 0.46690 |
| Gamma | 855.62 | -425.81 | 0.28270 | 0.00000 | 0.00100 | 0.04090 | 0.00150 | 0.32610 |
| Generalised gamma | 850.17 | -422.09 | 0.05110 | 0.00010 | 0.00290 | 0.01010 | 0.00130 | 0.06550 |
| Gompertz | 892.91 | -444.45 | 0.13220 | 0.00070 | 0.07880 | 0.01290 | 0.00230 | 0.22690 |
| Lognormal | 848.75 | -422.38 | 0.02280 | 0.00020 | 0.00610 | 0.00730 | 0.00450 | 0.04090 |
| Log-logistic | 845.89 | -420.95 | 0.05330 | 0.00010 | 0.00040 | 0.00840 | 0.00570 | 0.06790 |
| Generalised F | 843.77 | -417.88 | 0.00410 | 0.01030 | 0.03340 | 0.00110 | 0.01020 | 0.05910 |
| ***Fractional polynomial model*** |  |  |  |  |  |  |  |  |
| *First-order FP (Power 1)* |  |  |  |  |  |  |  |  |
| -2 | 120.08 | -58.04 | NR | 0.57917 | 0.38182 | 18.99812 | 90.02591 | NC |
| -1 | 118.85 | -57.42 | NR | 0.58252 | 0.38083 | 19.09938 | 90.52187 | NC |
| -0.5 | 123.99 | -59.99 | NR | 0.50784 | 0.12547 | 14.61903 | 74.58565 | NC |
| 0.5 | 149.08 | -72.54 | 4.34543 | 0.00630 | 3.67138 | 8.20660 | 10.40644 | 26.63616 |
| 1 | 160.14 | -78.07 | 1.31004 | 0.00628 | 1.40466 | 4.41007 | 6.05702 | 13.18808 |
| 2 | 169.59 | -82.80 | 0.33112 | 0.00347 | 0.10124 | 0.00027 | 0.10498 | 0.54107 |
| 3 | 171.02 | -83.51 | 0.37876 | 0.00905 | 0.11511 | 0.17934 | 0.81288 | 1.49514 |
| 0 | 135.23 | -65.61 | 1.60132 | 0.00107 | 0.00787 | 0.56759 | 0.69222 | 2.87006 |
| *Second-order FP (Power 1, Power 2)* |  |  |  |  |  |  |  |  |
| -2,-1 | 120.32 | -57.16 | NR | 0.58466 | 0.39290 | 19.24913 | 91.01157 | NC |
| -2,-0.5 | 120.64 | -57.32 | NR | 0.56149 | 0.30208 | 17.84795 | 86.07383 | NC |
| -2,0.5 | 121.83 | -57.91 | NR | 0.59418 | 0.44691 | 19.92536 | 93.25569 | NC |
| -2,1 | 122.08 | -58.04 | NR | 0.57993 | 0.38467 | 19.03911 | 90.17019 | NC |
| -2,2 | 121.36 | -57.68 | NR | 0.57279 | 0.35559 | 18.60441 | 88.64408 | NC |
| -2,3 | 120.35 | -57.17 | NR | 0.57279 | 0.35559 | 18.60441 | 88.64219 | NC |
| -1,-0.5 | 119.95 | -56.97 | NR | 0.62052 | 0.55594 | 21.49141 | 98.79760 | NC |
| -1,0.5 | 118.63 | -56.31 | NR | 0.49805 | 0.10132 | 14.05563 | 72.53756 | NC |
| -1,1 | 117.59 | -55.80 | NR | 0.53587 | 0.20640 | 16.26334 | 80.51290 | NC |
| -1,2 | 115.70 | -54.85 | NR | 0.55716 | 0.27880 | 17.52832 | 85.01390 | NC |
| -1,3 | 114.95 | -54.48 | NR | 0.56600 | 0.31216 | 18.06746 | 86.91643 | NC |
| -0.5,0.5 | 116.78 | -55.39 | NR | 0.27702 | 0.37440 | 3.29308 | 29.27782 | NC |
| -0.5,1 | 115.56 | -54.78 | NR | 0.32573 | 0.14637 | 5.25365 | 38.16643 | NC |
| -0.5,2 | 114.19 | -54.10 | NR | 0.40250 | 0.00285 | 8.87017 | 53.00566 | NC |
| -0.5,3 | 114.58 | -54.29 | NR | 0.43868 | 0.00827 | 10.74911 | 60.25949 | NC |
| 0.5,1 | 115.53 | -54.77 | 4.64217 | 0.00630 | 3.79231 | 8.39908 | 10.62305 | 27.46291 |
| 0.5,2 | 119.03 | -56.51 | 4.68536 | 0.00630 | 3.82314 | 8.44493 | 10.67526 | 27.63499 |
| 0.5,3 | 124.27 | -59.14 | 4.72875 | 0.00630 | 3.88084 | 8.53175 | 10.77219 | 27.91984 |
| 1,2 | 124.55 | -59.28 | 4.05849 | 0.00630 | 3.38040 | 7.76245 | 9.90551 | 25.11314 |
| 1,3 | 132.35 | -63.17 | 3.00873 | 0.00630 | 2.53697 | 6.41719 | 8.37814 | 20.34732 |
| 2,3 | 147.50 | -70.75 | 0.24460 | 0.00628 | 0.50181 | 2.45272 | 3.71334 | 6.91875 |
| -2,-2 | 120.41 | -57.20 | NR | 0.60440 | 0.47584 | 20.44924 | 95.22634 | NC |
| -1,-1 | 120.20 | -57.10 | 210.69773 | 0.59572 | 0.44065 | 19.93340 | 93.39866 | 325.06616 |
| -0.5,-0.5 | 119.16 | -56.58 | 4.81613 | 0.00630 | 3.98116 | 8.68137 | 10.94089 | 28.42586 |
| 0.5,0.5 | 115.00 | -54.50 | 4.72875 | 0.00630 | 3.84703 | 8.48101 | 10.71581 | 27.77890 |
| 1,1 | 117.70 | -55.85 | 4.64217 | 0.00630 | 3.80634 | 8.41996 | 10.64718 | 27.52195 |
| 2,2 | 136.89 | -65.45 | 0.55438 | 0.00628 | 0.66274 | 2.87512 | 4.22920 | 8.32772 |
| 3,3 | 158.10 | -76.05 | 1.11393 | 0.00099 | 0.02415 | 0.22790 | 0.42863 | 1.79559 |
| -2,0 | 121.23 | -57.62 | NR | 0.62874 | 0.61061 | 22.09642 | 100.76944 | NC |
| -1,0 | 119.44 | -56.72 | NR | 0.42241 | 0.00098 | 9.92114 | 57.05232 | NC |
| -0.5,0 | 118.06 | -56.03 | NR | 0.27776 | 0.37135 | 3.31489 | 29.38722 | NC |
| 0.5,0 | 115.44 | -54.72 | NR | 0.76445 | 1.40426 | 30.91675 | 130.64486 | NC |
| 1,0 | 114.76 | -54.38 | NR | 0.70431 | 1.09813 | 27.31404 | 118.07427 | NC |
| 2,0 | 115.27 | -54.64 | NR | 0.24082 | 0.08656 | 6.46982 | 34.89263 | NC |
| 3,0 | 117.79 | -55.90 | 0.97107 | 0.00048 | 0.01221 | 0.30491 | 0.50424 | 1.79292 |
| 0,0 | 116.62 | -55.31 | 0.57068 | 0.00187 | 0.00069 | 0.27561 | 0.55681 | 1.40565 |
| ***Parameter mixture model*** |  |  |  |  |  |  |  |  |
| Gengamma+Lognormal | 772.78 |  | 0.02560 | 0.00044 | 0.00531 | 0.00368 | 0.00743 | 0.04247 |
| Log-logistic+Lognormal | 788.39 | -389.19 | 0.02560 | 0.00046 | 0.00587 | 0.00220 | 0.00599 | 0.04012 |
| Gamma+Lognormal | 788.73 | -389.37 | 0.02560 | 0.00047 | 0.00599 | 0.00178 | 0.00563 | 0.03946 |
| Log-logistic+Log-logistic | 790.77 | -390.39 | 0.02560 | 0.00048 | 0.00083 | 0.00805 | 0.02019 | 0.05515 |
| Gengamma+Gengamma | 813.62 | -399.81 | 0.43560 | 0.00003 | 0.00073 | 0.00009 | 0.00005 | 0.43650 |
| Lognormal+Lognormal | 841.67 | -415.84 | 0.43560 | 0.00005 | 0.00059 | 0.00106 | 0.00027 | 0.43757 |
| Gengamma+Log-logistic | 842.87 | -415.43 | 0.43560 | 0.00000 | 0.00016 | 0.00062 | 0.00086 | 0.43724 |
| Gamma+Gengamma | 844.02 | -416.01 | 0.43560 | 0.00002 | 0.00050 | 0.00003 | 0.00002 | 0.43616 |
| Lognormal+Weibull | 845.8 | -417.90 | 0.43560 | 0.00003 | 0.00035 | 0.00127 | 0.00032 | 0.43757 |
| Gengamma+Weibull | 847.17 | -417.58 | 0.43560 | 0.00001 | 0.00007 | 0.00002 | 0.00009 | 0.43579 |
| Gompertz+Log-logistic | 847.77 | -418.89 | 0.43560 | 0.00022 | 0.00001 | 0.00500 | 0.00790 | 0.44874 |
| Weibull+Weibull | 848.04 | -419.02 | 0.43560 | 0.00001 | 0.00040 | 0.00112 | 0.00125 | 0.43837 |
| Exponential+Gamma | 848.05 | -420.02 | 0.43560 | 0.00000 | 0.00057 | 0.00046 | 0.00000 | 0.43663 |
| Gamma+Weibull | 848.13 | -419.06 | 0.43560 | 0.00001 | 0.00030 | 0.00110 | 0.00081 | 0.43782 |
| Gompertz+Weibull | 848.16 | -419.08 | 0.43560 | 0.00001 | 0.00040 | 0.00097 | 0.00141 | 0.43838 |
| Exponential+Weibull | 848.23 | -419.11 | 0.43560 | 0.00000 | 0.00001 | 0.00024 | 0.00030 | 0.43615 |
| Log-logistic+Weibull | 848.23 | -420.12 | 0.43560 | 0.00001 | 0.00020 | 0.00067 | 0.00004 | 0.43651 |
| Gamma+Log-logistic | 848.3 | -419.15 | 0.43560 | 0.00000 | 0.00026 | 0.00106 | 0.00110 | 0.43802 |
| Gamma+Gamma | 848.32 | -419.16 | 0.43560 | 0.00001 | 0.00029 | 0.00100 | 0.00103 | 0.43793 |
| Gamma+Gompertz | 848.34 | -419.17 | 0.43560 | 0.00001 | 0.00030 | 0.00105 | 0.00112 | 0.43808 |
| Exponential+Gengamma | 849.83 | -419.91 | 0.43560 | 0.00000 | 0.00016 | 0.00000 | 0.00021 | 0.43597 |
| Exponential+Log-logistic | 849.88 | -420.94 | 0.43560 | 0.00013 | 0.00006 | 0.01433 | 0.01186 | 0.46197 |
| Gengamma+Gompertz | 849.91 | -418.96 | 0.43560 | 0.00000 | 0.00004 | 0.00086 | 0.00108 | 0.43758 |
| Exponential+Lognormal | 852.33 | -422.16 | 0.02560 | 0.00024 | 0.00612 | 0.00697 | 0.00722 | 0.04615 |
| Gompertz+Lognormal | 854.22 | -422.11 | 0.02560 | 0.00020 | 0.00514 | 0.00618 | 0.00596 | 0.04307 |
| Exponential+Gompertz | 859.83 | -425.92 | 0.43560 | 0.00007 | 0.00307 | 0.00430 | 0.00086 | 0.44390 |
| Gompertz+Gompertz | 859.89 | -424.94 | 0.43560 | 0.00016 | 0.00020 | 0.00007 | 0.00061 | 0.43663 |
| Exponential+Exponential | 904.99 | -449.49 | 0.02560 | 0.00314 | 0.25331 | 0.00527 | 0.00638 | 0.29370 |
| ***Generalised additive model*** |  |  |  |  |  |  |  |  |
| 1 knot | 121.86 | -57.93 | 1.58760 | 0.00255 | 0.01963 | 0.00218 | 0.01636 | 1.62831 |
| 2 knots | 121.26 | -56.68 | 1.58760 | 0.00223 | 0.01651 | 0.00906 | 0.03534 | 1.65075 |
| 3 knots | 120.21 | -55.68 | 0.10890 | 0.00209 | 0.01729 | 0.00920 | 0.04060 | 0.17808 |
| 4 knots | 119.54 | -55.03 | 1.58760 | 0.00202 | 0.01595 | 0.00885 | 0.04528 | 1.65971 |
| 5 knots | 83.51 | -34.78 | 1.58760 | 0.00187 | 0.00023 | 0.07431 | 0.17548 | 1.83949 |
| 6 knots | 86.48 | -35.51 | 1.58760 | 0.00177 | 0.00099 | 0.06360 | 0.14470 | 1.79866 |
| 7 knots | 88.08 | -35.37 | 1.58760 | 0.00182 | 0.00057 | 0.06781 | 0.15658 | 1.81438 |
| 8 knots | 87.11 | -34.77 | 1.58760 | 0.00183 | 0.00059 | 0.06662 | 0.15928 | 1.81592 |
| 9 knots | 86.78 | -34.19 | 0.10890 | 0.00179 | 0.00094 | 0.06295 | 0.15848 | 0.33306 |
| 10 knots | 86.75 | -34.00 | 0.10890 | 0.00179 | 0.00182 | 0.05504 | 0.14892 | 0.31647 |
| ***Restricted cubic model*** |  |  |  |  |  |  |  |  |
| 1 knot | 121.84 | -57.92 | 0.02582 | 0.02877 | 4.58828 | 6.89710 | 8.43380 | 19.97377 |
| 2 knots | 121.12 | -56.56 | 0.02475 | 0.02896 | 4.59831 | 6.95298 | 8.53239 | 20.13740 |
| 3 knots | 120.76 | -55.38 | 0.02340 | 0.02838 | 4.59325 | 6.94040 | 8.56354 | 20.14898 |
| 4 knots | 120.53 | -54.26 | 0.02320 | 0.02835 | 4.61307 | 6.93952 | 8.61672 | 20.22085 |
| 5 knots | 82.77 | -34.39 | 0.02699 | 0.03265 | 4.67598 | 7.10348 | 8.76747 | 20.60657 |
| ***RP spline model*** |  |  |  |  |  |  |  |  |
| *Hazard* |  |  |  |  |  |  |  |  |
| 1 knot | 850.76 | -422.38 | 0.04000 | 0.00014 | 0.00330 | 0.00731 | 0.00179 | 0.05254 |
| 2 knots | 846.26 | -419.13 | 0.09000 | 0.00006 | 0.00004 | 0.00063 | 0.00030 | 0.09103 |
| 3 knots | 845.46 | -417.73 | 0.16000 | 0.00000 | 0.00015 | 0.00003 | 0.00087 | 0.16106 |
| *Odds* |  |  |  |  |  |  |  |  |
| 1 knot | 848.6 | -420.3 | 0.09000 | 0.00001 | 0.00013 | 0.00428 | 0.00002 | 0.09444 |
| 2 knots | 848.6 | -420.3 | 0.04000 | 0.00007 | 0.00005 | 0.00420 | 0.00200 | 0.04632 |
| 3 knots | 843.26 | -416.63 | 0.16000 | 0.00000 | 0.00004 | 0.00121 | 0.00290 | 0.16415 |
| *Normal* |  |  |  |  |  |  |  |  |
| 1 knot | 849.43 | -421.72 | 0.09000 | 0.00002 | 0.00132 | 0.01107 | 0.00047 | 0.10288 |
| 2 knots | 847.66 | -419.83 | 0.01000 | 0.00013 | 0.00121 | 0.00125 | 0.00022 | 0.01282 |
| 3 knots | 841.87 | -415.93 | 0.25000 | 0.00000 | 0.00025 | 0.00025 | 0.00130 | 0.25180 |

AIC: Akaike’s information criterion; NR: Not reached; MSE: Mean squared error; PFS: Progression-free survival; SP: Survival probability; "m": month; RMST: Restricted mean survival time; NC: Not be calculated; "-": The fitting procedure failed

Table S2. The selection of best model for overall survival curve in chemotherapy group

|  | ***AIC*** | ***Log Likelihood*** | ***MSE of median OS*** | ***MSE of 12-m SP*** | ***MSE of 6-m RMST*** | ***MSE of 12-m RMST*** | ***MSE of 18-m RMST*** | ***Cumulative MSE*** |
| --- | --- | --- | --- | --- | --- | --- | --- | --- |
| ***Standard parametric model*** |  |  |  |  |  |  |  |  |
| Exponential | 866.56 | -432.28 | 0.96100 | 0.00410 | 0.10920 | 0.52530 | 0.82550 | 2.42510 |
| Weibull | 846.35 | -421.17 | 0.03630 | 0.00020 | 0.00040 | 0.00390 | 0.00070 | 0.04150 |
| Gamma | 846.76 | -421.38 | 0.03100 | 0.00050 | 0.00070 | 0.00060 | 0.00890 | 0.04170 |
| Generalised gamma | 848.30 | -421.15 | 0.04890 | 0.00010 | 0.00020 | 0.00540 | 0.00000 | 0.05460 |
| Gompertz | 849.19 | -422.60 | 0.16530 | 0.00000 | 0.00400 | 0.00000 | 0.00000 | 0.16930 |
| Lognormal | 851.37 | -423.69 | 0.13940 | 0.00190 | 0.00070 | 0.01020 | 0.07440 | 0.22660 |
| Log-logistic | 848.70 | -422.35 | 0.08630 | 0.00070 | 0.00160 | 0.00110 | 0.00890 | 0.09860 |
| Generalised F | 850.31 | -421.15 | 0.04890 | 0.00010 | 0.00020 | 0.00540 | 0.00000 | 0.05460 |
| ***Fractional polynomial model*** |  |  |  |  |  |  |  |  |
| *First-order FP (Power 1)* |  |  |  |  |  |  |  |  |
| -2 | 94.31 | -45.15 | NR | 0.07668 | 0.01002 | 0.50835 | 12.28886 | NC |
| -1 | 91.55 | -43.78 | NR | 0.07339 | 0.01701 | 0.42145 | 11.57868 | NC |
| -0.5 | 90.63 | -43.31 | NR | 0.06371 | 0.05054 | 0.20357 | 9.51134 | NC |
| 0.5 | 92.26 | -44.13 | 212.89710 | 0.49083 | 7.17656 | 53.41580 | 118.85695 | 392.83724 |
| 1 | 93.88 | -44.94 | 176.91644 | 0.49055 | 2.51448 | 37.60647 | 94.58639 | 312.11433 |
| 2 | 96.98 | -46.49 | 60.69958 | 0.39513 | 0.13170 | 2.55556 | 24.99254 | 88.77451 |
| 3 | 99.91 | -47.95 | 3.09410 | 0.00002 | 0.15055 | 0.36507 | 0.00000 | 3.60974 |
| 0 | 90.93 | -43.47 | 1.56002 | 0.00000 | 0.04302 | 0.07841 | 0.05228 | 1.73372 |
| *Second-order FP (Power 1, Power 2)* |  |  |  |  |  |  |  |  |
| -2,-1 | 93.55 | -43.77 | NR | 0.07328 | 0.01727 | 0.41859 | 11.55488 | NC |
| -2,-0.5 | 92.20 | -43.10 | NR | 0.06652 | 0.03873 | 0.26019 | 10.10570 | NC |
| -2,0.5 | 89.72 | -41.86 | NR | 0.08089 | 0.00348 | 0.63328 | 13.22883 | NC |
| -2,1 | 89.03 | -41.52 | NR | 0.07891 | 0.00599 | 0.57455 | 12.79311 | NC |
| -2,2 | 88.79 | -41.40 | NR | 0.07734 | 0.00841 | 0.53055 | 12.45487 | NC |
| -2,3 | 89.52 | -41.76 | NR | 0.07695 | 0.00918 | 0.51853 | 12.36258 | NC |
| -1, -0.5 | 92.60 | -43.30 | NR | 0.06528 | 0.04385 | 0.23386 | 9.83541 | NC |
| -1,0.5 | 91.11 | -42.56 | NR | 0.07992 | 0.00467 | 0.60433 | 13.01872 | NC |
| -1,1 | 90.49 | -42.24 | NR | 0.07757 | 0.00808 | 0.53625 | 12.50080 | NC |
| -1,2 | 89.88 | -41.94 | NR | 0.07585 | 0.01128 | 0.48775 | 12.11908 | NC |
| -1,3 | 89.99 | -42.00 | NR | 0.07508 | 0.01288 | 0.46688 | 11.95117 | NC |
| -0.5,0.5 | 92.05 | -43.02 | NR | 0.07508 | 0.01284 | 0.46702 | 11.95048 | NC |
| -0.5,1 | 91.67 | -42.83 | NR | 0.07215 | 0.01980 | 0.39199 | 11.32152 | NC |
| -0.5,2 | 91.15 | -42.58 | NR | 0.06980 | 0.02670 | 0.33488 | 10.81454 | NC |
| -0.5,3 | 91.07 | -42.53 | NR | 0.06839 | 0.03155 | 0.30150 | 10.50438 | NC |
| 0.5,1 | 93.94 | -43.97 | 226.23149 | 0.49083 | 9.80009 | 60.41038 | 129.18490 | 426.11770 |
| 0.5,2 | 94.20 | -44.10 | 215.23806 | 0.49083 | 7.61379 | 54.63148 | 120.66701 | 398.64117 |
| 0.5,3 | 94.24 | -44.12 | 213.77346 | 0.49083 | 7.32357 | 53.82587 | 119.46826 | 394.88199 |
| 1,2 | 95.26 | -44.63 | 187.71723 | 0.49069 | 3.55025 | 41.84423 | 101.24492 | 334.84732 |
| 1,3 | 95.44 | -44.72 | 184.17187 | 0.49069 | 3.15243 | 40.34170 | 98.90011 | 327.05681 |
| 2,3 | 96.68 | -45.34 | 101.02248 | 0.48385 | 0.12391 | 10.53659 | 46.59501 | 158.76184 |
| -2,-2 | 95.35 | -44.67 | NR | 0.07585 | 0.01156 | 0.48635 | 12.11420 | NC |
| -1,-1 | 93.15 | -43.57 | NR | 0.07119 | 0.02293 | 0.36601 | 11.09987 | NC |
| -0.5,-0.5 | 92.60 | -43.30 | NR | 0.06072 | 0.06534 | 0.14968 | 8.88425 | NC |
| 0.5,0.5 | 93.56 | -43.78 | 230.46257 | 0.49083 | 10.60941 | 62.44652 | 132.15465 | 436.16399 |
| 1,1 | 94.63 | -44.31 | 202.23666 | 0.49069 | 5.56493 | 48.66892 | 111.71969 | 368.68090 |
| 2,2 | 96.33 | -45.17 | 102.63704 | 0.48371 | 0.13352 | 10.96478 | 47.48662 | 161.70568 |
| 3,3 | 97.10 | -45.55 | 1.34330 | 0.00032 | 0.09376 | 0.14252 | 0.04328 | 1.62317 |
| -2,0 | 90.82 | -42.41 | NR | 0.08381 | 0.00112 | 0.72265 | 13.87597 | NC |
| -1,0 | 91.87 | -42.93 | NR | 0.08324 | 0.00152 | 0.70373 | 13.74293 | NC |
| -0.5,0 | 92.40 | -43.20 | NR | 0.07981 | 0.00486 | 0.60030 | 12.98915 | NC |
| 0.5,0 | 92.92 | -43.46 | NR | 0.07328 | 0.00907 | 0.46688 | 11.80367 | NC |
| 1,0 | 92.91 | -43.46 | 93.72164 | 0.48024 | 0.07034 | 8.27604 | 41.60061 | 144.14888 |
| 2,0 | 92.74 | -43.37 | 15.61035 | 0.00953 | 0.04744 | 0.17708 | 2.23548 | 18.07988 |
| 3,0 | 92.65 | -43.33 | 1.66154 | 0.00005 | 0.04657 | 0.07829 | 0.06946 | 1.85591 |
| 0,0 | 92.77 | -43.39 | 1.61038 | 0.00001 | 0.04097 | 0.08527 | 0.03840 | 1.77502 |
| ***Parameter mixture model*** |  |  |  |  |  |  |  |  |
| Gengamma+Lognormal | 850.52 | -419.26 | 0.31360 | 0.00003 | 0.00006 | 0.00112 | 0.00239 | 0.31721 |
| Log-logistic+Lognormal | 849.13 | -419.57 | 0.31360 | 0.00001 | 0.00018 | 0.00124 | 0.00249 | 0.31753 |
| Gamma+Lognormal | - | - | - | - | - | - | - | - |
| Log-logistic+Log-logistic | 849.22 | -419.61 | 0.31360 | 0.00001 | 0.00017 | 0.00131 | 0.00215 | 0.31724 |
| Gengamma+Gengamma | 851.2 | -418.60 | 0.31360 | 0.00004 | 0.00284 | 0.13264 | 0.15148 | 0.60060 |
| Lognormal+Lognormal | 848.53 | -419.26 | 0.31360 | 0.00003 | 0.00009 | 0.00144 | 0.00265 | 0.31781 |
| Gengamma+Log-logistic | 850.65 | -419.32 | 0.31360 | 0.00005 | 0.00026 | 0.00116 | 0.00215 | 0.31722 |
| Gamma+Gengamma | - | - | - | - | - | - | - | - |
| Lognormal+Weibull | 848.69 | -419.35 | 0.31360 | 0.00001 | 0.00011 | 0.00187 | 0.00302 | 0.31862 |
| Gengamma+Weibull | 850.63 | -419.32 | 0.31360 | 0.00002 | 0.00004 | 0.00125 | 0.00259 | 0.31750 |
| Gompertz+Log-logistic | 854.92 | -422.46 | 0.31360 | 0.00002 | 0.00093 | 0.00401 | 0.00187 | 0.32043 |
| Weibull+Weibull | 849.3 | -419.65 | 0.31360 | 0.00000 | 0.00021 | 0.00100 | 0.00166 | 0.31648 |
| Exponential+Gamma | 850.76 | -421.38 | 0.31360 | 0.00031 | 0.00160 | 0.00341 | 0.00265 | 0.32157 |
| Gamma+Weibull | 849.51 | -419.75 | 0.31360 | 0.00000 | 0.00009 | 0.00117 | 0.00154 | 0.31640 |
| Gompertz+Weibull | 851.01 | -420.50 | 1.12360 | 0.00000 | 0.00003 | 0.00011 | 0.00067 | 1.12441 |
| Exponential+Weibull | 850.35 | -421.17 | 0.31360 | 0.00007 | 0.00121 | 0.00927 | 0.00034 | 0.32450 |
| Log-logistic+Weibull | 849.6 | -419.80 | 0.31360 | 0.00000 | 0.00017 | 0.00130 | 0.00174 | 0.31681 |
| Gamma+Log-logistic | 849.32 | -419.66 | 0.31360 | 0.00001 | 0.00017 | 0.00125 | 0.00234 | 0.31737 |
| Gamma+Gamma | 849.3 | -419.65 | 0.31360 | 0.00001 | 0.00010 | 0.00112 | 0.00214 | 0.31697 |
| Gamma+Gompertz | 850.92 | -420.46 | 1.12360 | 0.00002 | 0.00004 | 0.00027 | 0.00057 | 1.12449 |
| Exponential+Gengamma | 852.31 | -421.15 | 0.31360 | 0.00002 | 0.00085 | 0.01147 | 0.00227 | 0.32820 |
| Exponential+Log-logistic | 852.71 | -422.36 | 0.31360 | 0.00044 | 0.00276 | 0.00444 | 0.00248 | 0.32371 |
| Gengamma+Gompertz | 851.21 | -419.61 | 0.31360 | 0.09290 | 0.09847 | 2.73208 | 3.70024 | 6.93729 |
| Exponential+Lognormal | 854.55 | -423.27 | 0.31360 | 0.00065 | 0.00083 | 0.00025 | 0.01442 | 0.32975 |
| Gompertz+Lognormal | 849.79 | -419.90 | 0.31360 | 0.00089 | 0.00225 | 0.00023 | 0.01874 | 0.33571 |
| Exponential+Gompertz | 872.56 | -432.28 | 1.12360 | 0.00342 | 0.09929 | 0.47665 | 0.74736 | 2.45032 |
| Gompertz+Gompertz | 854.93 | -422.47 | 0.31360 | 0.00002 | 0.00093 | 0.00367 | 0.00170 | 0.31992 |
| Exponential+Exponential | 870.56 | -432.28 | 1.12360 | 0.00342 | 0.09929 | 0.47665 | 0.74753 | 2.45050 |
| ***Generalised additive model*** |  |  |  |  |  |  |  |  |
| 1 knot | 93.88 | -44.94 | 1.51290 | 0.00029 | 0.01454 | 0.00346 | 0.00070 | 1.53189 |
| 2 knots | - | - | - | - | - | - | - | - |
| 3 knots | - | - | - | - | - | - | - | - |
| 4 knots | - | - | - | - | - | - | - | - |
| 5 knots | - | - | - | - | - | - | - | - |
| 6 knots | - | - | - | - | - | - | - | - |
| 7 knots | - | - | - | - | - | - | - | - |
| 8 knots | - | - | - | - | - | - | - | - |
| 9 knots | - | - | - | - | - | - | - | - |
| 10 knots | - | - | - | - | - | - | - | - |
| ***Restricted cubic model*** |  |  |  |  |  |  |  |  |
| 1 knot | 95.56 | -44.78 | 0.02907 | 0.03907 | 0.18110 | 1.67444 | 7.31685 | 9.24053 |
| 2 knots | 97.15 | -44.58 | 0.02920 | 0.03872 | 0.15085 | 1.64000 | 7.22956 | 9.08834 |
| 3 knots | 94.25 | -42.12 | 0.02778 | 0.03819 | 0.10943 | 1.61707 | 6.84041 | 8.63289 |
| 4 knots | 95.98 | -41.99 | 0.02765 | 0.03809 | 0.10696 | 1.58342 | 6.77875 | 8.53486 |
| 5 knots | 97.64 | -41.82 | 0.02756 | 0.03801 | 0.10576 | 1.56453 | 6.73832 | 8.47418 |
| ***RP spline model*** |  |  |  |  |  |  |  |  |
| *Hazard* |  |  |  |  |  |  |  |  |
| 1 knot | 848.34 | -421.17 | 0.04000 | 0.00023 | 0.00046 | 0.00347 | 0.00105 | 0.04520 |
| 2 knots | 847.16 | -419.58 | 0.16000 | 0.00001 | 0.00001 | 0.00000 | 0.00000 | 0.16002 |
| 3 knots | 849.39 | -419.69 | 0.16000 | 0.00002 | 0.00002 | 0.00000 | 0.00000 | 0.16004 |
| *Normal* |  |  |  |  |  |  |  |  |
| 1 knot | 849.14 | -421.57 | 0.01000 | 0.00051 | 0.00068 | 0.00131 | 0.00829 | 0.02079 |
| 2 knots | 847.13 | -419.57 | 0.09000 | 0.00000 | 0.00007 | 0.00023 | 0.00000 | 0.09030 |
| 3 knots | 848.99 | -419.5 | 0.16000 | 0.00002 | 0.00002 | 0.00000 | 0.00001 | 0.16005 |
| *Odds* |  |  |  |  |  |  |  |  |
| 1 knot | 847.07 | -419.54 | 0.04000 | 0.00036 | 0.00075 | 0.00381 | 0.00243 | 0.04734 |
| 2 knots | 847.07 | -419.54 | 0.09000 | 0.00000 | 0.00001 | 0.00008 | 0.00001 | 0.09010 |
| 3 knots | 849.14 | -419.57 | 0.16000 | 0.00001 | 0.00001 | 0.00000 | 0.00001 | 0.16003 |

AIC: Akaike’s information criterion; NR: Not reached; MSE: Mean squared error; PFS: Progression-free survival; SP: Survival probability; "m": month; RMST: Restricted mean survival time; NC: Not be calculated; "-": The fitting procedure failed

Table S3. The selection of best model for progression-free survival curve in sacituzumab tirumotecan group

|  | ***AIC*** | ***Log Likelihood*** | ***MSE of median OS*** | ***MSE of 12-m SP*** | ***MSE of 6-m RMST*** | ***MSE of 12-m RMST*** | ***MSE of 18-m RMST*** | ***Cumulative MSE*** |
| --- | --- | --- | --- | --- | --- | --- | --- | --- |
| ***Standard parametric model*** |  |  |  |  |  |  |  |  |
| Exponential | 990.66 | -494.33 | 0.15120 | 0.00070 | 0.08150 | 0.10920 | 0.03760 | 0.38020 |
| Weibull | 982.26 | -489.13 | 0.07350 | 0.00050 | 0.00070 | 0.01600 | 0.01640 | 0.10710 |
| Gamma | 980.09 | -488.05 | 0.02060 | 0.00030 | 0.00250 | 0.01280 | 0.00960 | 0.04580 |
| Generalised gamma | 978.91 | -486.45 | 0.13320 | 0.00010 | 0.00000 | 0.00410 | 0.00200 | 0.13940 |
| Gompertz | 988.81 | -492.41 | 0.06080 | 0.00100 | 0.01450 | 0.00050 | 0.00210 | 0.07890 |
| Lognormal | 977.84 | -486.92 | 0.35380 | 0.00000 | 0.00070 | 0.02170 | 0.00860 | 0.38480 |
| Log-logistic | 979.73 | -487.87 | 0.16730 | 0.00000 | 0.00080 | 0.00310 | 0.00040 | 0.17160 |
| Generalised F | 980.92 | -486.46 | 0.13270 | 0.00010 | 0.00000 | 0.00410 | 0.00190 | 0.13880 |
| ***Fractional polynomial model*** |  |  |  |  |  |  |  |  |
| *First-order FP (Power 1)* |  |  |  |  |  |  |  |  |
| -2 | 129.34 | -62.67 | NR | 0.33062 | 0.07735 | 6.36701 | 47.72850 | NC |
| -1 | 130.08 | -63.04 | NR | 0.34504 | 0.10780 | 6.98489 | 50.53762 | NC |
| -0.5 | 131.42 | -63.71 | NR | 0.33675 | 0.08394 | 6.57766 | 48.78578 | NC |
| 0.5 | 135.91 | -65.96 | 29.42677 | 0.10440 | 3.93576 | 22.43354 | 38.66873 | 94.56919 |
| 1 | 137.67 | -66.84 | 15.40283 | 0.10427 | 0.78442 | 12.20389 | 24.75081 | 53.24621 |
| 2 | 139.63 | -67.82 | 0.01554 | 0.01565 | 0.06884 | 0.16185 | 1.80719 | 2.06907 |
| 3 | 140.49 | -68.24 | 0.61678 | 0.00420 | 0.07951 | 0.01374 | 0.25896 | 0.97319 |
| 0 | 133.61 | -64.80 | 1.11378 | 0.00326 | 0.00588 | 0.03385 | 0.49475 | 1.65152 |
| *Second-order FP (Power 1, Power 2)* |  |  |  |  |  |  |  |  |
| -2,-1 | 131.34 | -62.67 | NR | 0.33154 | 0.07926 | 6.40897 | 47.92214 | NC |
| -2,-0.5 | 131.32 | -62.66 | NR | 0.33420 | 0.08382 | 6.51523 | 48.42047 | NC |
| -2,0.5 | 131.25 | -62.62 | NR | 0.33258 | 0.08411 | 6.47598 | 48.17862 | NC |
| -2,1 | 131.25 | -62.63 | NR | 0.33270 | 0.08307 | 6.47140 | 48.18000 | NC |
| -2,2 | 131.32 | -62.66 | NR | 0.33131 | 0.07909 | 6.39935 | 47.86954 | NC |
| -2,3 | 131.33 | -62.67 | NR | 0.33039 | 0.07669 | 6.35440 | 47.67325 | NC |
| -1,-0.5 | 131.77 | -62.88 | NR | 0.34398 | 0.11037 | 6.98013 | 50.44667 | NC |
| -1,0.5 | 131.95 | -62.98 | NR | 0.33884 | 0.09032 | 6.68478 | 49.24084 | NC |
| -1,1 | 131.98 | -62.99 | NR | 0.34222 | 0.09975 | 6.84970 | 49.95495 | NC |
| -1,2 | 131.94 | -62.97 | NR | 0.34339 | 0.10325 | 6.90898 | 50.21114 | NC |
| -1,3 | 131.82 | -62.91 | NR | 0.34363 | 0.10370 | 6.91739 | 50.24941 | NC |
| -0.5,0.5 | 132.55 | -63.27 | NR | 0.29506 | 0.00936 | 4.68200 | 40.20932 | NC |
| -0.5,1 | 132.67 | -63.34 | NR | 0.31979 | 0.04517 | 5.78303 | 45.27112 | NC |
| -0.5,2 | 132.71 | -63.36 | NR | 0.32924 | 0.06509 | 6.21801 | 47.21039 | NC |
| -0.5,3 | 132.60 | -63.30 | NR | 0.33154 | 0.07045 | 6.32520 | 47.68292 | NC |
| 0.5,1 | 134.42 | -64.21 | 37.02290 | 0.10440 | 6.63720 | 28.57123 | 46.61364 | 118.94935 |
| 0.5,2 | 134.96 | -64.48 | 34.27686 | 0.10440 | 5.61015 | 26.33954 | 43.74921 | 110.08015 |
| 0.5,3 | 135.09 | -64.54 | 32.31518 | 0.10440 | 4.88444 | 24.69400 | 41.62209 | 103.62010 |
| 1,2 | 135.86 | -64.93 | 26.15959 | 0.10433 | 3.02786 | 20.06866 | 35.54328 | 84.90372 |
| 1,3 | 136.10 | -65.05 | 23.08461 | 0.10433 | 2.21021 | 17.71657 | 32.38883 | 75.50454 |
| 2,3 | 137.18 | -65.59 | 5.92749 | 0.10343 | 0.09017 | 5.23131 | 14.17838 | 25.53077 |
| -2,-2 | 131.31 | -62.65 | 4.64556 | 0.05857 | 0.04061 | 2.78454 | 0.16572 | 7.69499 |
| -1,-1 | 131.62 | -62.81 | 16.60273 | 0.10427 | 0.83134 | 12.75994 | 25.54108 | 55.83935 |
| -0.5,-0.5 | 132.06 | -63.03 | 34.74683 | 0.10440 | 5.84129 | 26.84593 | 44.40248 | 111.94092 |
| 0.5,0.5 | 133.91 | -63.95 | 37.02290 | 0.10440 | 6.60221 | 28.49858 | 46.52083 | 118.74891 |
| 1,1 | 135.11 | -64.55 | 29.86234 | 0.10440 | 4.01071 | 22.62150 | 38.91662 | 95.51555 |
| 2,2 | 136.83 | -65.42 | 6.42442 | 0.10324 | 0.10862 | 5.25192 | 14.20550 | 26.09369 |
| 3,3 | 137.57 | -65.79 | 1.13498 | 0.00264 | 0.02771 | 0.00759 | 0.31853 | 1.49145 |
| -2,0 | 131.27 | -62.64 | NR | 0.33663 | 0.09371 | 6.65998 | 49.00254 | NC |
| -1,0 | 131.88 | -62.94 | NR | 0.32535 | 0.05766 | 6.04862 | 46.43716 | NC |
| -0.5,0 | 132.33 | -63.17 | NR | 0.16557 | 0.36709 | 0.48761 | 15.88486 | NC |
| 0.5,0 | 133.24 | -63.62 | NR | 0.38825 | 0.26422 | 9.17480 | 59.73561 | NC |
| 1,0 | 133.55 | -63.77 | NR | 0.31002 | 0.08377 | 5.91946 | 44.88708 | NC |
| 2,0 | 133.81 | -63.91 | NR | 0.06076 | 0.00109 | 0.40704 | 8.22303 | NC |
| 3,0 | 133.80 | -63.90 | 0.57056 | 0.00176 | 0.00316 | 0.01904 | 0.37001 | 0.96453 |
| 0,0 | 132.82 | -63.41 | 0.42949 | 0.00220 | 0.00454 | 0.00897 | 0.37013 | 0.81532 |
| ***Parameter mixture model*** |  |  |  |  |  |  |  |  |
| Gengamma+Lognormal | 956.66 | -472.33 | 0.04840 | 0.00034 | 0.00000 | 0.00010 | 0.00121 | 0.05006 |
| Log-logistic+Lognormal | 955.61 | -472.80 | 0.04840 | 0.00016 | 0.00056 | 0.00000 | 0.00072 | 0.04984 |
| Gamma+Lognormal | 981.3 | -485.65 | 0.04840 | 0.00058 | 0.00003 | 0.00086 | 0.00367 | 0.05354 |
| Log-logistic+Log-logistic | 956.17 | -473.08 | 0.04840 | 0.00015 | 0.00055 | 0.00001 | 0.00063 | 0.04974 |
| Gengamma+Gengamma | 982.51 | -484.26 | 0.04840 | 0.00025 | 0.00009 | 0.00188 | 0.00126 | 0.05189 |
| Lognormal+Lognormal | 973.19 | -481.59 | 0.07840 | 0.00028 | 0.00011 | 0.00226 | 0.00005 | 0.08109 |
| Gengamma+Log-logistic | 954.02 | -471.01 | 0.04840 | 0.00019 | 0.00076 | 0.00015 | 0.00220 | 0.05171 |
| Gamma+Gengamma | 980.51 | -484.26 | 0.04840 | 0.00025 | 0.00010 | 0.00176 | 0.00125 | 0.05176 |
| Lognormal+Weibull | 980.67 | -485.34 | 0.04840 | 0.00058 | 0.00004 | 0.00207 | 0.00248 | 0.05357 |
| Gengamma+Weibull | 978.52 | -483.26 | 0.04840 | 0.00025 | 0.00008 | 0.00323 | 0.00148 | 0.05344 |
| Gompertz+Log-logistic | 963.23 | -476.62 | 0.07840 | 0.00077 | 0.00043 | 0.00008 | 0.01014 | 0.08981 |
| Weibull+Weibull | 985.19 | -487.59 | 0.04840 | 0.00081 | 0.00021 | 0.00166 | 0.00904 | 0.06013 |
| Exponential+Gamma | 982.52 | -487.26 | 0.04840 | 0.00009 | 0.00355 | 0.00223 | 0.00086 | 0.05513 |
| Gamma+Weibull | 983.98 | -486.99 | 0.04840 | 0.00079 | 0.00005 | 0.00093 | 0.00726 | 0.05743 |
| Gompertz+Weibull | 985.7 | -487.85 | 0.04840 | 0.00010 | 0.00386 | 0.00585 | 0.00242 | 0.06063 |
| Exponential+Weibull | 983.7 | -487.85 | 0.04840 | 0.00011 | 0.00389 | 0.00607 | 0.00262 | 0.06109 |
| Log-logistic+Weibull | 984.03 | -487.02 | 0.04840 | 0.00073 | 0.00000 | 0.00062 | 0.00491 | 0.05466 |
| Gamma+Log-logistic | 957.19 | -473.59 | 0.51840 | 0.00065 | 0.00054 | 0.01570 | 0.01548 | 0.55076 |
| Gamma+Gamma | 956.62 | -473.31 | 0.51840 | 0.00068 | 0.00057 | 0.01656 | 0.01724 | 0.55345 |
| Gamma+Gompertz | 984.53 | -487.26 | 0.04840 | 0.00009 | 0.00355 | 0.00226 | 0.00087 | 0.05517 |
| Exponential+Gengamma | 982.91 | -486.45 | 0.07840 | 0.00018 | 0.00037 | 0.00116 | 0.00004 | 0.08015 |
| Exponential+Log-logistic | 983.74 | -487.87 | 0.07840 | 0.00011 | 0.00181 | 0.00063 | 0.00032 | 0.08127 |
| Gengamma+Gompertz | 982.07 | -485.03 | 0.04840 | 0.00098 | 0.00076 | 0.00122 | 0.00282 | 0.05417 |
| Exponential+Lognormal | 981.85 | -486.92 | 0.07840 | 0.00015 | 0.00015 | 0.01362 | 0.00292 | 0.09524 |
| Gompertz+Lognormal | 980.51 | -485.26 | 0.04840 | 0.00102 | 0.00004 | 0.00075 | 0.00394 | 0.05416 |
| Exponential+Gompertz | 988.87 | -490.43 | 0.04840 | 0.00032 | 0.00019 | 0.00514 | 0.00158 | 0.05564 |
| Gompertz+Gompertz | 990.87 | -490.43 | 0.04840 | 0.00032 | 0.00020 | 0.00511 | 0.00158 | 0.05561 |
| Exponential+Exponential | 994.66 | -494.33 | 0.07840 | 0.00099 | 0.07225 | 0.08874 | 0.02347 | 0.26386 |
| ***Generalised additive model*** |  |  |  |  |  |  |  |  |
| 1 knot | 136.43 | -65.29 | 0.01440 | 0.00111 | 0.04836 | 0.02123 | 0.00044 | 0.08554 |
| 2 knots | 137.07 | -65.32 | 0.01440 | 0.00115 | 0.05144 | 0.02229 | 0.00037 | 0.08965 |
| 3 knots | 137.30 | -65.35 | 0.01440 | 0.00117 | 0.05336 | 0.02277 | 0.00043 | 0.09214 |
| 4 knots | 137.36 | -65.31 | 0.01440 | 0.00117 | 0.05336 | 0.02253 | 0.00044 | 0.09190 |
| 5 knots | 137.40 | -65.32 | 0.01440 | 0.00118 | 0.05364 | 0.02259 | 0.00044 | 0.09225 |
| 6 knots | 137.43 | -65.27 | 0.01440 | 0.00117 | 0.05322 | 0.02223 | 0.00042 | 0.09145 |
| 7 knots | 137.46 | -65.28 | 0.01440 | 0.00117 | 0.05345 | 0.02232 | 0.00043 | 0.09178 |
| 8 knots | 137.46 | -65.27 | 0.01440 | 0.00117 | 0.05341 | 0.02229 | 0.00043 | 0.09170 |
| 9 knots | 137.46 | -65.27 | 0.01440 | 0.00117 | 0.05341 | 0.02229 | 0.00043 | 0.09170 |
| 10 knots | 137.47 | -65.27 | 0.01440 | 0.00117 | 0.05341 | 0.02232 | 0.00043 | 0.09173 |
| ***Restricted cubic model*** |  |  |  |  |  |  |  |  |
| 1 knot | 136.33 | -65.16 | 0.04015 | 0.03581 | 1.03433 | 5.30185 | 11.26340 | 17.67554 |
| 2 knots | 137.87 | -64.94 | 0.03967 | 0.03532 | 0.96696 | 5.21191 | 11.03901 | 17.29287 |
| 3 knots | 139.03 | -64.51 | 0.03891 | 0.03447 | 0.94372 | 5.00195 | 10.76136 | 16.78042 |
| 4 knots | 140.18 | -64.09 | 0.04013 | 0.03488 | 0.96189 | 5.06523 | 10.88418 | 16.98632 |
| 5 knots | 140.27 | -63.13 | 0.03800 | 0.03372 | 0.87402 | 4.81764 | 10.47499 | 16.23837 |
| ***RP spline model*** |  |  |  |  |  |  |  |  |
| *Hazard* |  |  |  |  |  |  |  |  |
| 1 knot | 980.01 | -487.01 | 0.04000 | 0.00013 | 0.00026 | 0.00047 | 0.00000 | 0.04086 |
| 2 knots | 981.18 | -486.59 | 0.01000 | 0.00037 | 0.00016 | 0.00007 | 0.00066 | 0.01126 |
| 3 knots | 981.77 | -485.89 | 0.00000 | 0.00015 | 0.00014 | 0.00000 | 0.00016 | 0.00045 |
| Normal |  |  |  |  |  |  |  |  |
| 1 knot | 978.71 | -486.35 | 0.09000 | 0.00004 | 0.00006 | 0.00405 | 0.00233 | 0.09648 |
| 2 knots | 979.01 | -485.5 | 0.00000 | 0.00027 | 0.00045 | 0.00002 | 0.00010 | 0.00084 |
| 3 knots | 980.05 | -485.03 | 0.00000 | 0.00014 | 0.00019 | 0.00000 | 0.00001 | 0.00035 |
| *Odds* |  |  |  |  |  |  |  |  |
| 1 knot | 980.42 | -486.21 | 0.09000 | 0.00002 | 0.00093 | 0.00220 | 0.00050 | 0.09364 |
| 2 knots | 980.42 | -486.21 | 0.00000 | 0.00021 | 0.00026 | 0.00001 | 0.00003 | 0.00051 |
| 3 knots | 981.23 | -485.61 | 0.00000 | 0.00012 | 0.00018 | 0.00002 | 0.00000 | 0.00032 |

AIC: Akaike’s information criterion; NR: Not reached; MSE: Mean squared error; PFS: Progression-free survival; SP: Survival probability; "m": month; RMST: Restricted mean survival time; NC: Not be calculated; "-": The fitting procedure failed

Table S4. The selection of best model for overall survival curve in sacituzumab tirumotecan group

|  | ***AIC*** | ***Log Likelihood*** | ***MSE of median PFS*** | ***MSE of 12-m SP*** | ***MSE of 6-m RMST*** | ***MSE of 12-m RMST*** | ***MSE of 18-m RMST*** | ***Cumulative MSE*** |
| --- | --- | --- | --- | --- | --- | --- | --- | --- |
| ***Standard parametric model*** |  |  |  |  |  |  |  | - |
| Exponential | 635.80 | -316.90 | NR | 0.00030 | 0.03530 | 0.09650 | 0.10730 | 0.23940 |
| Weibull | 631.53 | -313.76 | NR | 0.00010 | 0.00100 | 0.00230 | 0.01010 | 0.01350 |
| Gamma | 630.86 | -313.43 | NR | 0.00020 | 0.00040 | 0.00330 | 0.00970 | 0.01360 |
| Generalised gamma | 631.66 | -312.83 | NR | 0.00040 | 0.00020 | 0.00010 | 0.00020 | 0.00090 |
| Gompertz | 634.99 | -315.50 | NR | 0.00010 | 0.01060 | 0.00580 | 0.00040 | 0.01690 |
| Lognormal | 629.88 | -312.94 | NR | 0.00020 | 0.00020 | 0.00010 | 0.00040 | 0.00090 |
| Log-logistic | 630.30 | -313.15 | NR | 0.00000 | 0.00040 | 0.00110 | 0.00210 | 0.00360 |
| Generalised F | 633.39 | -312.70 | NR | 0.00030 | 0.00000 | 0.00000 | 0.00020 | 0.00050 |
| ***Fractional polynomial model*** |  |  |  |  |  |  |  |  |
| *First-order FP (Power 1)* |  |  |  |  |  |  |  |  |
| -2 | 84.96 | -40.48 | NR | 0.03819 | 0.03954 | 0.06412 | 5.43420 | 5.57605 |
| -1 | 82.82 | -39.41 | NR | 0.03973 | 0.03311 | 0.08539 | 5.73907 | 5.89730 |
| -0.5 | 82.70 | -39.35 | NR | 0.03769 | 0.04358 | 0.05580 | 5.31642 | 5.45348 |
| 0.5 | 85.94 | -40.97 | NR | 0.61085 | 6.45443 | 56.57734 | 139.88890 | 203.53152 |
| 1 | 87.41 | -41.70 | NR | 0.61023 | 0.79042 | 30.72808 | 96.97845 | 129.10718 |
| 2 | 88.94 | -42.47 | NR | 0.01679 | 0.13672 | 0.60403 | 7.04034 | 7.79787 |
| 3 | 89.57 | -42.78 | NR | 0.00003 | 0.14551 | 0.41847 | 0.00352 | 0.56753 |
| 0 | 84.06 | -40.03 | NR | 0.00038 | 0.07299 | 0.15983 | 0.05159 | 0.28479 |
| *Second-order FP (Power 1, Power 2)* |  |  |  |  |  |  |  | - |
| -2,-1 | 84.03 | -39.01 | NR | 0.03989 | 0.03275 | 0.08709 | 5.76498 | 5.92470 |
| -2,-0.5 | 84.70 | -39.35 | NR | 0.03769 | 0.04366 | 0.05561 | 5.31319 | 5.45015 |
| -2,0.5 | 85.95 | -39.98 | NR | 0.03941 | 0.03271 | 0.08318 | 5.69412 | 5.84943 |
| -2,1 | 86.35 | -40.18 | NR | 0.03910 | 0.03492 | 0.07746 | 5.62371 | 5.77518 |
| -2,2 | 86.76 | -40.38 | NR | 0.03855 | 0.03785 | 0.06886 | 5.50342 | 5.64867 |
| -2,3 | 86.91 | -40.45 | NR | 0.03831 | 0.03891 | 0.06595 | 5.46080 | 5.60398 |
| -1,-0.5 | 84.63 | -39.32 | NR | 0.03855 | 0.03903 | 0.06740 | 5.49123 | 5.63620 |
| -1,0.5 | 84.82 | -39.41 | NR | 0.03989 | 0.03249 | 0.08744 | 5.76642 | 5.92624 |
| -1,1 | 84.82 | -39.41 | NR | 0.03961 | 0.03369 | 0.08341 | 5.71371 | 5.87042 |
| -1,2 | 84.75 | -39.38 | NR | 0.03957 | 0.03391 | 0.08278 | 5.70511 | 5.86137 |
| -1,3 | 84.69 | -39.35 | NR | 0.03961 | 0.03380 | 0.08312 | 5.70941 | 5.86594 |
| -0.5,0.5 | 84.10 | -39.05 | NR | 0.02767 | 0.12409 | 0.00396 | 3.33076 | 3.48646 |
| -0.5,1 | 83.98 | -38.99 | NR | 0.03295 | 0.07522 | 0.01002 | 4.35697 | 4.47516 |
| -0.5,2 | 83.91 | -38.95 | NR | 0.03539 | 0.05772 | 0.02928 | 4.84632 | 4.96871 |
| -0.5,3 | 83.92 | -38.96 | NR | 0.03623 | 0.05251 | 0.03768 | 5.01015 | 5.13657 |
| 0.5,1 | 83.96 | -38.98 | NR | 0.61101 | 11.23293 | 69.81256 | 160.30507 | 241.96157 |
| 0.5,2 | 84.72 | -39.36 | NR | 0.61101 | 9.70420 | 65.81900 | 154.22569 | 230.35991 |
| 0.5,3 | 85.28 | -39.64 | NR | 0.61101 | 7.77605 | 60.48781 | 146.00043 | 214.87530 |
| 1,2 | 85.70 | -39.85 | NR | 0.61085 | 4.78278 | 51.06519 | 131.14060 | 187.59943 |
| 1,3 | 86.44 | -40.22 | NR | 0.61085 | 3.33741 | 45.69072 | 122.43342 | 172.07240 |
| 2,3 | 88.00 | -41.00 | NR | 0.60167 | 0.13101 | 11.61508 | 59.25095 | 71.59871 |
| -2,-2 | 83.14 | -38.57 | NR | 0.04163 | 0.02571 | 0.11540 | 6.11838 | 6.30113 |
| -1,-1 | 84.42 | -39.21 | NR | 0.03922 | 0.03582 | 0.07690 | 5.62608 | 5.77801 |
| -0.5,-0.5 | 84.49 | -39.24 | NR | 0.55275 | 0.03019 | 1.27124 | 28.40318 | 30.25736 |
| 0.5,0.5 | 83.67 | -38.83 | NR | 0.61101 | 11.22757 | 69.79919 | 160.28481 | 241.92258 |
| 1,1 | 84.55 | -39.27 | NR | 0.61101 | 6.71461 | 57.37291 | 141.14067 | 205.83920 |
| 2,2 | 87.20 | -40.60 | NR | 0.60167 | 0.14437 | 12.50535 | 61.24258 | 74.49397 |
| 3,3 | 88.78 | -41.39 | NR | 0.00017 | 0.10802 | 0.25280 | 0.00525 | 0.36623 |
| -2,0 | 85.39 | -39.69 | NR | 0.04138 | 0.02451 | 0.11602 | 6.10602 | 6.28793 |
| -1,0 | 84.77 | -39.39 | NR | 0.04106 | 0.02718 | 0.10739 | 6.01542 | 6.19105 |
| -0.5,0 | 84.29 | -39.14 | NR | 0.00578 | 0.66595 | 1.06583 | 0.07056 | 1.80812 |
| 0.5,0 | 83.67 | -38.83 | NR | 0.04508 | 0.01335 | 0.18620 | 6.86511 | 7.10975 |
| 1,0 | 83.67 | -38.84 | NR | 0.04126 | 0.02207 | 0.12028 | 6.12531 | 6.30892 |
| 2,0 | 83.95 | -38.97 | NR | 0.01365 | 0.05748 | 0.02522 | 1.97272 | 2.06907 |
| 3,0 | 84.22 | -39.11 | NR | 0.00005 | 0.06308 | 0.16272 | 0.01998 | 0.24583 |
| 0,0 | 83.87 | -38.93 | NR | 0.00003 | 0.06551 | 0.19580 | 0.01251 | 0.27384 |
| ***Parameter mixture model*** |  |  |  |  |  |  |  | - |
| Gengamma+Lognormal | 629.72 | -308.86 | NR | 0.00000 | 0.00014 | 0.00009 | 0.00096 | 0.00120 |
| Log-logistic+Lognormal | 634.78 | -312.39 | NR | 0.00002 | 0.00007 | 0.00033 | 0.00153 | 0.00195 |
| Gamma+Lognormal | - | - | - | - | - | - | - | - |
| Log-logistic+Log-logistic | 627.95 | -308.97 | NR | 0.00002 | 0.00036 | 0.00004 | 0.00028 | 0.00070 |
| Gengamma+Gengamma | 631.07 | -308.54 | NR | 0.00003 | 0.00015 | 0.00023 | 0.00013 | 0.00054 |
| Lognormal+Lognormal | 634.96 | -312.48 | NR | 0.00009 | 0.00033 | 0.00197 | 0.00037 | 0.00277 |
| Gengamma+Log-logistic | 629.27 | -308.64 | NR | 0.00004 | 0.00023 | 0.00012 | 0.00001 | 0.00040 |
| Gamma+Gengamma | - | - | - | - | - | - | - | - |
| Lognormal+Weibull | 627.73 | -308.86 | NR | 0.00000 | 0.00011 | 0.00009 | 0.00120 | 0.00141 |
| Gengamma+Weibull | 629.12 | -308.56 | NR | 0.00003 | 0.00015 | 0.00017 | 0.00016 | 0.00050 |
| Gompertz+Log-logistic | 635.59 | -312.80 | NR | 0.00005 | 0.00006 | 0.00194 | 0.00071 | 0.00277 |
| Weibull+Weibull | 627.86 | -308.93 | NR | 0.00000 | 0.00008 | 0.00005 | 0.00150 | 0.00163 |
| Exponential+Gamma | - | - | - | - | - | - | - | - |
| Gamma+Weibull | 627.71 | -308.85 | NR | 0.00000 | 0.00011 | 0.00008 | 0.00126 | 0.00145 |
| Gompertz+Weibull | 628.12 | -309.06 | NR | 0.00000 | 0.00004 | 0.00003 | 0.00179 | 0.00186 |
| Exponential+Weibull | 633.81 | -312.91 | NR | 0.00003 | 0.00003 | 0.00317 | 0.00171 | 0.00494 |
| Log-logistic+Weibull | 628.01 | -309.00 | NR | 0.00000 | 0.00015 | 0.00007 | 0.00122 | 0.00144 |
| Gamma+Log-logistic | - | - | - | - | - | - | - |  |
| Gamma+Gamma | 627.76 | -308.88 | NR | 0.00001 | 0.00017 | 0.00021 | 0.00026 | 0.00065 |
| Gamma+Gompertz | 629.74 | -309.87 | NR | 0.00001 | 0.00100 | 0.00493 | 0.00011 | 0.00605 |
| Exponential+Gengamma | 634.15 | -312.08 | NR | 0.00036 | 0.00048 | 0.00009 | 0.00368 | 0.00463 |
| Exponential+Log-logistic | 633.59 | -312.80 | NR | 0.00005 | 0.00006 | 0.00193 | 0.00068 | 0.00272 |
| Gengamma+Gompertz | 637.49 | -312.75 | NR | 0.00004 | 0.00005 | 0.00163 | 0.00089 | 0.00262 |
| Exponential+Lognormal | 633.02 | -312.51 | NR | 0.00010 | 0.00032 | 0.00166 | 0.00023 | 0.00230 |
| Gompertz+Lognormal | 635.07 | -312.53 | NR | 0.00009 | 0.00037 | 0.00217 | 0.00048 | 0.00312 |
| Exponential+Gompertz | 641.8 | -316.90 | NR | 0.00028 | 0.02969 | 0.07890 | 0.08509 | 0.19396 |
| Gompertz+Gompertz | 638.5 | -314.25 | NR | 0.00000 | 0.00105 | 0.00113 | 0.00071 | 0.00289 |
| Exponential+Exponential | 639.8 | -316.90 | NR | 0.00028 | 0.02969 | 0.07890 | 0.08509 | 0.19396 |
| ***Generalised additive model*** |  |  |  |  |  |  |  |  |
| 1 knot | 86.22 | -40.19 | NR | 0.00021 | 0.00740 | 0.00162 | 0.00007 | 0.00930 |
| 2 knots | 86.60 | -39.93 | NR | 0.00021 | 0.00626 | 0.00161 | 0.00001 | 0.00809 |
| 3 knots | 86.44 | -39.46 | NR | 0.00021 | 0.00493 | 0.00096 | 0.00002 | 0.00612 |
| 4 knots | 86.68 | -39.58 | NR | 0.00021 | 0.00546 | 0.00117 | 0.00001 | 0.00685 |
| 5 knots | 86.74 | -39.58 | NR | 0.00021 | 0.00552 | 0.00119 | 0.00000 | 0.00692 |
| 6 knots | 86.77 | -39.57 | NR | 0.00021 | 0.00554 | 0.00120 | 0.00000 | 0.00694 |
| 7 knots | 86.78 | -39.56 | NR | 0.00021 | 0.00557 | 0.00121 | 0.00000 | 0.00699 |
| 8 knots | 86.81 | -39.58 | NR | 0.00021 | 0.00563 | 0.00124 | 0.00000 | 0.00707 |
| 9 knots | 86.83 | -39.58 | NR | 0.00021 | 0.00566 | 0.00125 | 0.00000 | 0.00712 |
| 10 knots | 86.84 | -39.59 | NR | 0.00021 | 0.00567 | 0.00126 | 0.00000 | 0.00714 |
| ***Restricted cubic model*** |  |  |  |  |  |  |  |  |
| 1 knot | 86.12 | -40.06 | NR | 0.03074 | 0.09385 | 1.04439 | 4.73591 | 5.90489 |
| 2 knots | 86.98 | -39.49 | NR | 0.03036 | 0.06430 | 1.02398 | 4.61463 | 5.73328 |
| 3 knots | 84.44 | -37.22 | NR | 0.02954 | 0.04738 | 0.98249 | 4.35832 | 5.41772 |
| 4 knots | 85.02 | -36.51 | NR | 0.02940 | 0.04850 | 0.93132 | 4.29746 | 5.30668 |
| 5 knots | 86.61 | -36.31 | NR | 0.02940 | 0.04763 | 0.93182 | 4.34026 | 5.34910 |
| ***RP spline model*** |  |  |  |  |  |  |  |  |
| *Hazard* |  |  |  |  |  |  |  |  |
| 1 knot | 631.48 | -312.74 | NR | 0.00004 | 0.00009 | 0.00000 | 0.00003 | 0.00017 |
| 2 knots | 633.20 | -312.60 | NR | 0.00001 | 0.00027 | 0.00019 | 0.00001 | 0.00048 |
| 3 knots | 635.39 | -312.70 | NR | 0.00002 | 0.00019 | 0.00004 | 0.00000 | 0.00026 |
| Odds |  |  |  |  |  |  |  |  |
| 1 knot | *633.03* | -312.51 | NR | 0.00005 | 0.00006 | 0.00001 | 0.00008 | 0.00020 |
| 2 knots | 633.03 | -312.51 | NR | 0.00001 | 0.00024 | 0.00005 | 0.00005 | 0.00035 |
| 3 knots | 635.24 | -312.62 | NR | 0.00002 | 0.00016 | 0.00006 | 0.00000 | 0.00023 |
| *Normal* |  |  |  |  |  |  |  |  |
| 1 knot | 631.72 | -312.86 | NR | 0.00001 | 0.00019 | 0.00012 | 0.00012 | 0.00045 |
| 2 knots | 633.71 | -312.86 | NR | 0.00001 | 0.00021 | 0.00011 | 0.00016 | 0.00049 |
| 3 knots | 635.30 | -312.65 | NR | 0.00002 | 0.00005 | 0.00001 | 0.00007 | 0.00015 |

AIC: Akaike’s information criterion; NR: Not reached; MSE: Mean squared error; PFS: Progression-free survival; SP: Survival probability; "m": month; RMST: Restricted mean survival time; NC: Not be calculated; "-": The fitting procedure faile

# 13. Model structure

Figure S73. Model structure

# 14. The best model

Figure S74. Optimal fitting model for PFS curves

Figure S75. Optimal fitting model for OS curves

# 15. The reconstructed curve

Figure S76. The reconstructed PFS curve

Figure S77. The reconstructed OS curve

# 16. Distrubution of bootstrap sampling for survival curve

Figure S78. Distrubution of progression-free survival curve for chemotherapy group

Figure S79. Distrubution of overall survival curve for chemotherapy group

Figure S80. Distrubution of progression-free survival curve for sacituzumab tirumotecan group

Figure S81. Distrubution of progression-free survival curve for sacituzumab tirumotecan group

# 17. The reuslts of 1,000 bootstrap sampling for survival curve

Figure S82. Progression-free survival curve for chemotherapy group in 1,000 bootstrap sampling

Figure S83. Progression-free survival curve for chemotherapy group in 1,000 bootstrap sampling

Figure S83. Progression-free survival curve for sacituzumab tirumotecan group in 1,000 bootstrap sampling

Figure S85. Progression-free survival curve for sacituzumab tirumotecan group in 1,000 bootstrap sampling

# 18. CHEERS 2022 Checklist

# Table S5. CHEERS 2022 Checklist

| **Topic** | **No.** | **Item** | **Location where item is reported** |
| --- | --- | --- | --- |
| **Title** |  |  |  |
|  | 1 | Identify the study as an economic evaluation and specify the interventions being compared. | Title, |
| **Abstract** |  |  |  |
|  | 2 | Provide a structured summary that highlights context, key methods, results, and alternative analyses. | Abstract, Page 1 |
| **Introduction** |  |  |  |
| **Background and objectives** | 3 | Give the context for the study, the study question, and its practical relevance for decision making in policy or practice. | Introduction, Pages 1-5 |
| **Methods** |  |  |  |
| **Health economic analysis plan** | 4 | Indicate whether a health economic analysis plan was developed and where available. | Not applicable |
| **Study population** | 5 | Describe characteristics of the study population (such as age range, demographics, socioeconomic, or clinical characteristics). | Methods, Page 6 |
| **Setting and location** | 6 | Provide relevant contextual information that may influence findings. | Methods, Pages 11-12 |
| **Comparators** | 7 | Describe the interventions or strategies being compared and why chosen. | Methods, Page 6 |
| **Perspective** | 8 | State the perspective(s) adopted by the study and why chosen. | Methods, Page 7 |
| **Time horizon** | 9 | State the time horizon for the study and why appropriate. | Methods, Page 9 |
| **Discount rate** | 10 | Report the discount rate(s) and reason chosen. | Methods, Page 10 |
| **Selection of outcomes** | 11 | Describe what outcomes were used as the measure(s) of benefit(s) and harm(s). | Methods, Page 10 |
| **Measurement of outcomes** | 12 | Describe how outcomes used to capture benefit(s) and harm(s) were measured. | Methods, Page 10 |
| **Valuation of outcomes** | 13 | Describe the population and methods used to measure and value outcomes. | Methods, Page 10 |
| **Measurement and valuation of resources and costs** | 14 | Describe how costs were valued. | Methods  Page 11 |
| **Currency, price date, and conversion** | 15 | Report the dates of the estimated resource quantities and unit costs, plus the currency and year of conversion. | Methods, Page 10 |
| **Rationale and description of model** | 16 | If modelling is used, describe in detail and why used. Report if the model is publicly available and where it can be accessed. | Methods, Page 10 |
| **Analytics and assumptions** | 17 | Describe any methods for analysing or statistically transforming data, any extrapolation methods, and approaches for validating any model used. | Methods , Pages 7-9 |
| **Characterising heterogeneity** | 18 | Describe any methods used for estimating how the results of the study vary for subgroups. | Methods , Pages 12 |
| **Characterising distributional effects** | 19 | Describe how impacts are distributed across different individuals or adjustments made to reflect priority populations. | Not applicable |
| **Characterising uncertainty** | 20 | Describe methods to characterise any sources of uncertainty in the analysis. | Methods , Pages 12 |
| **Approach to engagement with patients and others affected by the study** | 21 | Describe any approaches to engage patients or service recipients, the general public, communities, or stakeholders (such as clinicians or payers) in the design of the study. | Not applicable |
| **Results** |  |  |  |
| **Study parameters** | 22 | Report all analytic inputs (such as values, ranges, references) including uncertainty or distributional assumptions. | Table 1 |
| **Summary of main results** | 23 | Report the mean values for the main categories of costs and outcomes of interest and summarise them in the most appropriate overall measure. | Table 1 |
| **Effect of uncertainty** | 24 | Describe how uncertainty about analytic judgments, inputs, or projections affect findings. Report the effect of choice of discount rate and time horizon, if applicable. | Results, Pages 13-14 |
| **Effect of engagement with patients and others affected by the study** | 25 | Report on any difference patient/service recipient, general public, community, or stakeholder involvement made to the approach or findings of the study | Not applicable |
| **Discussion** |  |  |  |
| **Study findings, limitations, generalisability, and current knowledge** | 26 | Report key findings, limitations, ethical or equity considerations not captured, and how these could affect patients, policy, or practice. | Discussion, Pages 14-15 |
| **Other relevant information** |  |  |  |
| **Source of funding** | 27 | Describe how the study was funded and any role of the funder in the identification, design, conduct, and reporting of the analysis | End of manuscript |
| **Conflicts of interest** | 28 | Report authors conflicts of interest according to journal or International Committee of Medical Journal Editors requirements. | End of manuscript |

*From:* Husereau D, Drummond M, Augustovski F, et al. Consolidated Health Economic Evaluation Reporting Standards 2022 (CHEERS 2022) Explanation and Elaboration: A Report of the ISPOR CHEERS II Good Practices Task Force. Value Health 2022;25. <doi:10.1016/j.jval.2021.10.008>
